# Heat Shock Protein HSP24 Is Involved in the BABA-Induced Resistance to Fungal Pathogen in Postharvest Grapes Underlying an NPR1-Dependent Manner

**DOI:** 10.3389/fpls.2021.646147

**Published:** 2021-03-08

**Authors:** Chunhong Li, Shifeng Cao, Kaituo Wang, Changyi Lei, Nana Ji, Feng Xu, Yongbo Jiang, Linglan Qiu, Yonghua Zheng

**Affiliations:** ^1^College of Life and Food Engineering, Chongqing Three Gorges University, Chongqing, China; ^2^College of Food Science and Technology, Nanjing Agricultural University, Nanjing, China; ^3^College of Biological and Environmental Sciences, Zhejiang Wanli University, Ningbo, China; ^4^College of Food and Pharmaceutical Sciences, Ningbo University, Ningbo, China

**Keywords:** β-aminobutyric acid, heat shock protein, priming resistance, NPR1, *Botrytis cinerea*, grape berries

## Abstract

Although heat shock proteins (HSPs), a family of ubiquitous molecular chaperones, are well characterized in heat stress-related responses, their function in plant defense remains largely unclear. Here, we report the role of *VvHSP24*, a class B HSP from *Vitis vinifera*, in β-aminobutyric acid (BABA)-induced priming defense against the necrotrophic fungus *Botrytis cinerea* in grapes. Grapes treated with 10 mmol L^–1^ BABA exhibited transiently increased transcript levels of *VvNPR1* and several SA-inducible genes, including *PR1*, *PR2*, and *PR5*. Additionally, phytoalexins accumulated upon inoculation with the gray mold fungus *B. cinerea*, which coincided with the action of a priming mode implicated in pathogen-driven resistance. Intriguingly, electrophoretic mobility shift (EMSA), yeast two-hybrid (Y2H) and His pull-down assays demonstrated that the nuclear chaperone *VvHSP24* cannot modulate the transcript of *PR* genes but does directly interact with *VvNPR1 in vivo* or *in vitro*. Furthermore, we found that *VvHSP24* overexpression enhanced the transcript levels of NPR1 and SA-responsive genes (*PR1*, *PR2*, and *PR5*) and increased the resistance of transgenic *Arabidopsis thaliana* to *B. cinerea* compared with wildtype Col-0. An opposite trend between CRISPR mutants of *AtHSFB1* (the orthologous gene of *VvHSP24* in *Arabidopsis*) and wildtype plants was observed. Hence, our results suggest that *VvHSP24* has a potential role in NPR1-dependent plant resistance to fungal pathogen. BABA-induced priming defense in grapes may require posttranslational modification of the chaperone *VvHSP24* to activate *VvNPR1* transcript, leading to *PR* gene expressions and resistance phenotypes.

## Introduction

Grapes are cultivated worldwide with great functional importance in terms of their attractive flavor and constitution of natural phytonutrients (Dai et al., 2011). Nevertheless, the shelf life of postharvest grape berries is very limited, which is mainly ascribed to the invasion of bacterial and fungal pathogens. Indeed, gray mold disease by the fungal pathogen *Botrytis cinerea* has been recognized as one of the most severe epidemics threatening economic effectiveness in fruit crop farming (Feliziani et al., 2013). A variety of *B. cinerea* strains can secrete mycotoxins and extracellular cell wall-degrading enzymes to disintegrate and digest the host cell walls of vine crops (Gabler et al., 2003). Ongoing application of synthetic fungicides for controlling gray mold rot of grapevines is highly efficiently, but the occurrences of resistance as well as the negative impacts posed by the frequent use of fungicides have gradually threatened the environment. Hence, eco-compatible alternatives for harmful fungicides to suppress the postharvest spoilage of grapes have been urgently demanded (Mari et al., 2014).

Among a wide spectrum of emerging approaches, several phytohormones and a few organic elicitors have been described as high effectiveness against disease pressure in different kinds of agronomic fruits (Mengiste et al., 2009; Burketova et al., 2015; Romanazzi et al., 2016). To optimize defense against invading pathogens, plants gradually potentiate the systemic immunity driven by stress-induced signaling. This response has been defined as a “priming defense,” which suggests that systemic reactions against pathogen invasions can be triggered (van Hulten et al., 2006). β-aminobutyric acid (BABA), a non-proteinogenic amino acid, has been well documented as a predominant priming elicitor for effective resistance induction and is very desirable for disease management in agricultural fields (Cohen et al., 2016; Baccelli et al., 2017). The biobased resistance inducer of BABA employs its defense functions via abscisic acid (ABA)-responsive and/or phosphoinositide-responsive signaling transductions (Ton et al., 2005). A series of studies utilizing various *Arabidopsis* defense pathway mutants revealed that BABA not only primes the expressions of *AtPR1*, *AtPR2*, and *AtPR5* through a salicylic acid (SA) signaling pathway but also intensifies the transcripts of a set of jasmonate acid (JA)- or ethylene (ET)-inducible genes, including *AtPDFs*, *AtVSPs*, and *AtHELs* (Zimmerli et al., 2001; Ton et al., 2002). Hence, BABA-induced resistance (BABA-IR) has been considered as an integrated disease alternative in plants and can be regulated by a signaling crosstalk involving SA, ABA, PI, JA, ET, and other hormone signaling pathways, which is dependent on the stress category (Cohen et al., 2016; AbuQamar et al., 2017; Sham et al., 2019). Similarly, the recent studies concluded that BABA-IR in peach, strawberry and grape occurs in a dose/concentration-dependent manner as a disease-dependent priming defense responding to BABA concentrations ranging from 10 to 50 mmol L^–1^, in contrast to a direct defense triggered by relatively high concentrations (≥100 mmol L^–1^ BABA) (Wang et al., 2016, 2018, 2019; Li et al., 2020b). Of note, there are metabolic costs involved in the activation of a certain defense, regardless of what type of BABA-IR is elicited (Perazzolli et al., 2011; Luna et al., 2014). Although the priming defense has some obvious ecological advantages by reducing the excessive cost of cellular resources unless plants suffer from severe hostile pressures (van Hulten et al., 2006), the critical node and its function underlying a priming defense have not been characterized in the specific plant-fungus interactions.

Biotic and abiotic stresses are environmental constraints that differ from natural or optimal conditions, impeding normal cellular functions and constituting a major hindrance to plant growth and development (Uyttewaal et al., 2010). Heat shock proteins (HSPs) are ubiquitous molecular chaperones that are recruited under diverse stressful conditions, including heat stress and other non-heat environmental stresses, such as osmotic, drought, salt, ultraviolet (UV) light, oxidative stresses, and pathogen infections (Swindell et al., 2007). HSPs/chaperones may serve as hubs in the proteostasis network by assisting in protein folding, protein assembly, compartment targeting, degradation, and signaling (Baniwal et al., 2004; Swindell et al., 2007). It has been evident that the expression of HSPs is constitutively regulated at the transcriptional level by heat shock transcription factors (HSFs), which are the terminal components of a signal transduction pathway modulating the transcript of genes responsive to distinct stimuli (von Koskull-Döring et al., 2007; Xin et al., 2010). *Arabidopsis* contains 21 HSF representatives that have been classified into three classes (A, B, and C) based on their characteristic cluster of DNA-binding domains (DBDs) and adjacent oligomerization domains (ODs, HR-A/B regions) (Nover et al., 2001; Scharf et al., 2012). Class B and Class C HSFs are characterized by the absence of the typical activator motif aromatic hydrophobic acidic (AHA), which is essential for the transcriptional activity of Class A HSFs (Guo et al., 2016). Along this line, HSF1a, HSF1b, HSF1d, and HSF1e are all responsible for basal thermotolerance, and the heat-acclimation phenotype fundamentally involves HSFA2, HSFA3, and HSFA7a (Bharti et al., 2000; Nishizawa et al., 2006; Larkindale and Vierling, 2008; Zheng et al., 2016). Expression of *AtHSFA4a* is transcriptionally elicited by numerous stresses, such as cold, drought and UV-B, and transgenic *Arabidopsis* overexpressing HSFA4a showed enhanced tolerance to those stresses (Pérez-Salamó et al., 2014). Enhancements of *AtHSFA2* and *AtHSFA7a* transcripts have been monitored during virus infection, revealing the involvement of these two HSFs in the disease resistance of *Arabidopsis* (Sugio et al., 2009). Moreover, overexpression of HSP27 strongly augmented plant resistance to various apoptotic stimulis (Samali et al., 2001; Tanabe et al., 2010), and the multichaperone complex HSP70-HSP90 is implicated in resistance protein (R protein)-mediated immunity and is essential for proper defense signal transduction (Noël et al., 2007; Shirasu, 2009; Li et al., 2012).

For decades, HSFs/HSPs have been recognized as hallmarks of the environmental stress response, and research on most HSFs/HSPs has typically focused on their central roles in response to several abiotic stresses, in particular heat stress management, in model plants such as *Arabidopsis thaliana*, peruvian tomato (*Solanum peruvianum*), rice (*Oryza sativa*), and wheat (*Triticum aestivum*). Nonetheless, much less is known about the functional modulation of HSFs/HSPs against biotic stresses, especially against fungal pathogens, in horticultural crops. In this research, our focus extends the exploration of the chaperone function and potential regulation of HSFs/HSPs involved in the BABA-elicited priming response to fungal infection in grape fruit.

## Materials and Methods

### Plant and Fungi

Grapes (*Vitis vinifera* Cv ‘Kyoho’) from 6-year-old vines were purchased and hand picked from a local supplier with a standard orchard in Chuanshan District, Suining City, Sichuan Province, China. The grapes coincided with some of reliable quality parameters, including the berry diameter of 1.8–2.2 cm, total soluble solids (SCC) of 13.4–15.7%, titratable acidity (TA) of 0.64–0.72%, pH value of 3.14–3.27 and firmness of 4.7–6.3 N cm^–2^ were late-harvested at a commercial maturity stage that was identified according to the Balic et al. (2014) and Rosales et al. (2016). The harvested grapes were transported to our laboratory within 1 h by an air-condition vehicle (20°C).

*Arabidopsis thaliana* wildtype (WT) Columbia-0 (Col-0) plants were applied for genetic transformation, and WT and transgenic *Arabidopsis* lines were grown in a SPT-P500B illumination incubator (Darth carter Co., Hefei, China) equipped with an automatic stable controller for maintaining the R.H. at 60–70%, illumination at 2500–3000 lx and temperature at 22°C with a long-day photoperiod (light/dark cycle of 14 h/10 h) over 4 weeks.

*Botrytis cinerea* (Pers.: Fr.) B05.10 strain (phylogenetic analysis shown in Supplementary Figure 1) was isolated from decayed ‘Kyoho’ grape berries (Lovato et al., 2019) and cultured on PDA medium (Haibo Co., Qingdao, China) for 14 days at 26°C. The cultured medium was then rinsed with sterile water containing 0.5% (v/v) Tween-80 to produce *B. cinerea* suspensions and diluted to a final concentration of 1.0 × 10^5^ spores mL^–1^, as counted with a Neubauer chamber.

### Grape Treatments

Intact grape berries were picked and partitioned into four lots of 360 grapes each, which were superficially sterilized by 75% (v/v) alcohol and dried on sterile filter paper at 20°C for 2 h. Two symmetrical holes were then wounded (1.5-mm-deep × 1.5-mm-diameter) at the equator of each grape with a dissecting needle. BABA (purity ≥ 99%, Sigma Co., St. Louis, MO, United States), at 10 mmol L^–1^, was selected as the most effective concentration in basis of our current findings (Wang et al., 2019, 2020). Grapes in each lot were exposed to the four treatments following the description of Porat et al. (2003): (1) control, berries solely mock-inoculated with sterile water; (2) BABA, berries solely elicited with BABA solution; (3) inoculation, berries solely challenged with the *B. cinerea* suspensions; and (4) BABA + inoculation, berries elicited with BABA and subsequently challenged with *B. cinerea* suspensions. All treated grapes were then ventilated on stainless steel mesh for 6 h, transferred to PE boxes (60-μm-thick walls) and arranged in incubators for 5 days under 20 ± 1°C and 85 ± 5% R.H. A total of 30 g tissues from the healthy sarcocarp (3 mm distant from the diseased holes) of 60 grapes in each lot were sliced by a sterile scalpel before the incubation (0 h) and at 1-day intervals during the incubation. Tissues taken from each individual were rapidly frozen in liquid N_2_ and kept at –80°C until analysis. Each treatment was performed according to a completely randomized design and repeated three times, and the experiment was performed twice.

### Assessment of Disease Development

If the width of the dot-inoculated area was beyond 1.5 mm, the grape could be defined to be diseased. Percentage of decaying grapes was calculated as disease incidence. The disease development of twenty berries from each triplicate was monitored continuously for 5 days at the intervals of 2 days.

### Measurement of Phytoalexin Content

For phytoalexin extraction, approximately 5 g of frozen pulp was ground in liquid N_2_ and homogenized with 25 mL of 85% (v/v) methanol in ice bath. The homogenates were transferred into a dark incubator at 4°C overnight and centrifuged at 10,000 × *g* for 15 min (4°C). The supernatants were retained and evaporated under nitrogen, and the residue was further dissolved in absolute methanol before filtration. The individual phytoalexin content in the prepared extractions was measured by a HPLC analysis as described by Vitrac et al. (2005).

### Classification and Sequence Analysis of HSFs and HSPs

Annotated complete lists of HSFs and HSPs in *Vitis vinifera* and *Arabidopsis thaliana* were obtained from the NCBI database. Furthermore, the phylogenetic relationship among the 37 identified *Vitis vinifera* HSFs/HSPs (*VvHSFs*/*VvHSPs*) and 20 *Arabidopsis thaliana* HSFs (*AtHSFs*) was conducted using MEGA 5.0 software with the neighbor-joining (NJ) method with 1000 bootstrap replicates and further visualized via the iTOL webtool. In addition, the online web resources SMART (Letunic and Bork, 2018) and TBtools (Chen et al., 2020) were used to visualize the conserved domains and sequence conservation, respectively.

### RNA Isolation and Real-Time qRT-PCR

Aliquots (5 g) of frozen tissues were ground in liquid N_2_, and total RNA was extracted using RNAprep Pure Kit for plants (Tiangen, China). Aliquots (1 μg) of RNA were prepared to synthesize first-strand cDNA using a PrimeScript^TM^ RT reagent kit (Takara, Japan). Quantitative real-time PCR (qRT-PCR) was conducted in triplicate using an ABI 7500 Fast Real-Time PCR system (Applied Biosystems). The 10-μL qPCR mixture contained 0.2 μL of each individual 10 μmol L^–1^ primer for *VvHSP24*, SA-dependent genes including *VvNPR1*, *VvPR1*, *VvPR2*, and *VvPR5* as well as JA/ET-dependent genes including *VvPDF1.2*, *VvVSP1*, *VvHEL*, *VvFAD3*, *VvTHI2*, *VvERS1*, and *VvERF1* (listed in Supplementary Table 1), 1 μL cDNA template, 5 μL 2 × ChamQ Universal SYBR qPCR Master Mix (Vazyme, China) and 3.6 μL ddH_2_O. The thermal cycle for qRT-PCR was 95°C for 30 s, followed by 40 cycles of 95°C for 10 s and 60°C for 30 s with the ABI-specified hold and melt curve stages. Amplicon lengths were optimized to 90–150 bp to ensure optimal polymerization efficiency. qRT-PCR results were normalized by the cycle threshold value (CT) using the reference gene *VvActin7* or *AtActin2* according to the 2*^–Δ^ ^Δ^ ^*CT*^* method (Livak and Schmittgen, 2001). Relative expression levels of *HSP/HSF* and *PR* genes were calibrated, with the values for the control fruit/plant being set as 1.

### Subcellular Localization

PCR-amplified *VvHSP24* was cloned into the pEAQ-GFP vector to construct the pEAQ-*VvHSP24*-GFP fusion protein and the pEAQ-GFP empty vector was used as the positive control. Then, onion bulb epidermis cells were transformed by inoculation with *A. tumefaciens* strain EHA105 harboring pEAQ-GFP and the recombinant construction of pEAQ-*VvHSP24*-GFP via an *Agrobacterium*-mediated system, respectively. Briefly, the adaxial epidermises obtained from onion bulb were cultured on Murashige and Skoog (MS) medium for approximately 1 week and then immersed overnight in the suspension of EHA105 cells containing pEAQ-*VvHSP24*-GFP or pEAQ-GFP, respectively. Following 3 days of incubation on MS medium at 26°C in darkness, these epidermises were stained with the nuclear dye 4,6-diamidino-phenylindole (DAPI, 20 μg mL^–1^, Sigma, United States). Then, the fluorescence signal of the stained onion peels was captured by a Zeiss LSM 510 confocal laser scanning microscope with excitation wavelength set at 405 nm for DAPI fluorescence and 488 nm for GFP fluorescence and emission wavelength in the range of 450–550 nm at 3 h after DAPI staining.

### Y2H Assay

Y2H assay was performed by employing the Matchmaker^TM^ Gold Y2H System (Clontech). Full-length cDNA clones of *VvHSP24* and *VvNPR1* in BABA-treated, *B. cinerea*-infected berries were inserted into the pGBKT7 and pGADT7 plasmids, respectively. The recombinant plasmids, including BD-*VvHSP24* and AD-*VvNPR1*, were cotransformed into yeast strain AH109 cells, which were further smeared on double dropout medium (DDO, SD/-Leu-Trp) and cultured for about 3 days at 29°C (Li et al., 2020b). Transformed colonies were cultured on triple dropout medium (TDO, SD/-Leu-Trp-His) and quadruple dropout medium (QDO, SD/-Leu-Trp-His-Ade) with or without 40 mg L^–1^ X-α-gal to determine possible interaction.

### His Pull-Down Assay

The recombinant GST-*VvNPR1* and His-*VvHSP24* proteins were obtained through fusing with pGEX-4T-1 or pET-28a vector, respectively. The recombinant proteins were transformed into *Escherichia coli* BL21 (DE3) cells and expressed at 20°C for 3 h after the addition of 0.1 mmol L^–1^ IPTG (isopropyl β-D-galactopyranoside). Bacteria expressing the GST-*VvNPR1* fusion protein were lysed by sonication and purified using glutathione agarose (Thermo Fisher Scientific, United States) following the instructions. *E. coli* harboring the His-*VvHSP24* fusion protein were isolated and purified using a B-PER (R) 6 × His Spin Purification Kit (Pierce, Rockford, IL, United States). For His pull-down assay, the purified His-*VvHSP24* fusion protein was immobilized with Ni sepharose 6 Fast Flow (GE Healthcare, code number: 17-5318-01) and mixed for 2 h at 4°C. Then, 5 g of GST-*VvNPR1* fusion protein was incubated with 15 μL of prewashed resin overnight (4°C) before being washed three times with cold phosphate-buffered saline (PBS) based on standard protocols. Pulled down proteins (6 μL) were boiled in 2× Laemmli buffer and detected by Western blotting with an anti-GST antibody (TransGen Biotech Co., Ltd., Beijing, China, HT601); the His + GST-*VvNPR1* fusion protein was used as a negative control.

### EMSA Assay

The CDS fragment of *VvHSP24* was synthesized and cloned into the pCzn1 vector to produce the His-*VvHSP24* recombinant protein and introduced into ArcticExpress (DE3) chemically competent cells. Then, 0.5 mmol L^–1^ IPTG was applied to induce the expression of the target recombinant protein (His-*VvHSP24*) at 15°C for approximately 9 h and then purified using a Ni-NTA His-Bind resin column (Novagen, Gibbstown, NJ, United States). The heat stress elements (HSE: 5′-GAAnnTTC-3′), which could be recognized by HSFs/HSPs, were detected in the promoters of *PR1*, *PR2* and *PR5* (Supplementary Data 1), and their nucleotides adjacent to HSF motifs (approximately 50 bp) were adopted as probes, respectively. These probes were labeled at the 5′ end with biotin using Light Shift chemiluminescent EMSA kit (Pierce Biotechnology, Rockford, IL, United States) and incubated with the purified His-*VvHSP24*. The protein-DNA complexes formed during EMSA experiments were transferred electrophoretically to a nitrocellulose membrane after separated by SDS-PAGE. Then, the shift bands were visualized via a chemiluminescence reaction on X-ray films. The addition of 200-fold mutant probes with the mutant HSF motif served as the competitors for the 5′-labeled WT DNA probes to test for the specific of the binding assays; the biotin-EBNA control DNA + EBNA extract was used as a positive control.

### Overexpression and Analysis of *Vitis vinifera* HSP24 in Transgenic *Arabidopsis* Plant

The coding region of *VvHSP24* was amplified by PCR technology from a grape berry cDNA library with gene-specific primer listed in Supplementary Table 1. The construction of a plasmid for overexpression of *VvHSP24* by introducing the *VvHSP24* fragment into the binary vector pCambia 1305.1 under the control of the cauliflower mosaic virus (CaMV) 35S promoter. *Arabidopsis* plant was transformed with *A. tumefaciens* GV3101 harboring the resulting *35S:VvHSP24* construct using the floral dip method (Clough and Bent, 1998). The harvested T1 seeds were surface sterilized in 2% (v/v) sodium hypochlorite for 10 min and stratification at 4°C for 3 days and then germinated on MS medium containing 50 mg L^–1^ kanamycin (kan) for more than 7 days. The kan-resistant seedlings were sown in pots containing a 1: 2 perlite-vermiculite mixture and cultured in illumination incubator with long-day photoperiod. Ultimately, homozygous plants of the T3 generation were used for subsequent experiments.

### Generation of HSFB1 Mutants by CRISPR-Cas9

Mutations were introduced into *Arabidopsis* HSFB1 through the non-homologous end joining (NHEJ) method of RNA-guided endonuclease-mediated targeted mutagenesis with the clustered regularly interspersed short palindromic repeats (CRISPR)-Cas9 system (Lowder et al., 2015; Li et al., 2019). In brief, the gene-targeting binary vector M2CRISPR, with specific guide RNA (sgRNA) cassettes under the control of the U26:U29 promoter as well as an EC promoter driving Cas9 expression, was provided by Shanghai Weidi Biotechnology Co., Ltd. sgRNA cassettes were designed using sequences corresponding to the protospacer adjacent motif (PAM) sites (set as ‘NGG’) on the sense or antisense strand of the targeted *AtHSFB1* gene, and the web server CRISPRdirect^1^ was used to check their off-target effects. Then, two sgRNA cassettes were annealed, and PCR products were further ligated into the *Bsa*I-digested vector M2CRISPR to generate a U26 promoter-driven T1 sgRNA and U29 promoter-driven T2 sgRNA (Supplementary Figure 4). The primers used are provided in Supplementary Table 1. The resulting binary vector was used for *A. tumefaciens* GV3101-mediated genetic transformation of *Arabidopsis*. T1 generation transformants were selected on MS medium containing 50 mg L^–1^ hygromycin phosphotransferase (HYG), and the effectiveness of genome editing was evaluated using the HYG-resistant seedlings. Genomic DNA from true leaves was isolated, and the targeted genomic region was amplified by PCR using KOD FX (Toyobo Co. Ltd., Osaka, Japan) with the primers listed in Supplementary Table 1. The resulting PCR products were sequenced, and mutants were identified by comparison with WT sequences. INDELs (insertions and deletions) at the targeted sites were considered as mutations. The T1 mutant seedlings were transplanted into soil, and T2 generation seeds were collected. After the identification of homozygous mutants, the T2 seeds were used to produce T3 seedlings.

### Inoculation of *Arabidopsis* Plant With Fungal Pathogen *B. cinerea*

Four- to five-week-old T3 transgenic and wildtype (Col-0) *Arabidopsis* were dot-inoculated with *B. cinerea* conidial suspensions (1.0 × 10^5^ conidia mL^–1^) as previously described (Xiao and Chye, 2011). Detached leaf samples were used for morphological observations at 6 dpi and leaves picked at 0, 3, and 6 dpi were used to test for the transcript levels of disease resistance-related genes.

### Trypan Blue Staining

Trypan blue staining was employed to assess the rate of cell death and fungal development of *B. cinerea*-infiltrated leaves from *Arabidopsis* WT (Col-0), *VvHSP24*-overexpressing plants and CRISPR mutants at 6 dpi, as described previously, with minor modifications (Cai et al., 2020). *B. cinerea*-infected *Arabidopsis* leaves were immersed and boiled in trypan blue staining solution [0.025% (m/v) trypan blue, 25% (m/v) phenol, 25% (v/v) lactic acid, and 25% (v/v) glycerol] for 5 min and then allowed to cool naturally and soak overnight. The stained leaves were immersed in 1.25 g mL^–1^ chloral hydrate solution for more than 24 h to eliminate chlorophyll; the samples were observed by binocular stereomicroscopy (Leica EZ4D) and photographed.

### Electrolyte Leakage Measurement

Estimation of electrolyte leakage was carried out as previously described (Mackey et al., 2002). Leaf disks (8 mm diameter) of *B. cinerea*-infiltrated leaves from WT and *VvHSP24*-overexpressing *Arabidopsis* were washed in 50 mL ddH_2_O at least twice and then transferred to 10 mL ddH_2_O and floated for 8 h at 120 rpm and 25°C. Water conductivity was detected over time using a conductivity meter (MIK-TDS210-B).

### Statistical Analysis

All data were subjected to *t*-test and one-way analysis of variance according to a Tukey’s test using SAS version 8.2 (SAS Inst., NC, United States); data were reported as the mean ± standard error of three replicates from one independent experiment. Different letters and asterisks indicated that the differences were statistically significant at the 0.05 or 0.01 level of the *p*-value.

## Results

### Inhibitory Effectiveness of BABA Treatment Against *B. cinerea* Infection in Grapes

**FIGURE 1:**
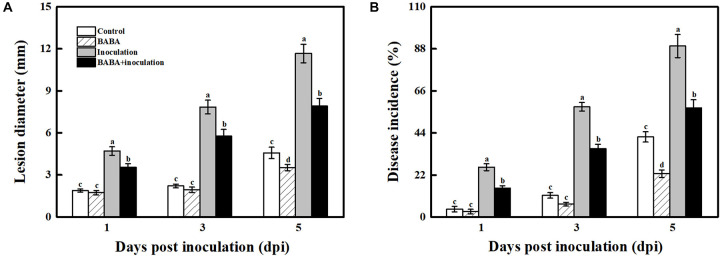
Impact of BABA on the disease development of grapes altered in their responses to the fungal pathogen *B. cinerea*. Lesion diameter **(A)** and disease incidence **(B)** measured at 1, 3, and 5 days post inoculation with *B. cinerea*. Grapes were pretreated with water (gray bars) or BABA (black bars) before inoculation. Disease development is expressed as the mean ± SE of nine assessments. Different letters above the bar indicate a significant difference (*p* < 0.05) between grapes with BABA-treated or *B. cinerea*-inoculated and untreated cases.

As presented in Figure 1, grape berries are obviously susceptible to the necrotrophic pathogen *B. cinerea* and became deteriorated after infection at 20°C. 10 mmol L^–1^ BABA treatment caused a significant inhibition in disease development in postharvest grapes, and their lesion diameter and disease incidence were 32.16 and 32.32%, respectively, lower compared with the only *B. cinerea*-inoculated fruit at 5 dpi (Figure 1 and Supplementary Figure 2).

### Molecular Identification and Characterization of *VvHSP24*

**FIGURE 2:**
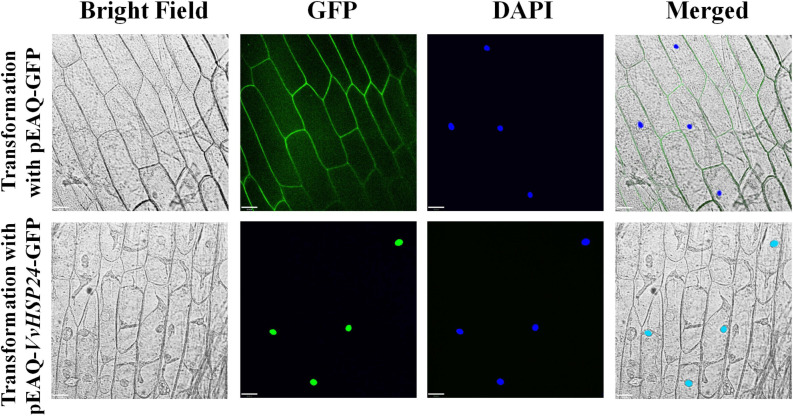
Nuclear localization of pEAQ-*VvHSP24*-GFP fusion protein. The fluorescence signals of pEAQ-GFP and pEAQ-*VvHSP24*-GFP fusion protein were observed with onion peels after transformation by *Agrobacterium tumefaciens*. Nuclei were visualized by staining onion peels with 4,6-diamino-2-phenylindole (DAPI), and GFP signals were captured using LSM (bars = 70 μm).

Given that *AtHSFB1*—a class B-HSF from *Arabidopsis*, also referred to as *AtHSF4* or *AtTBF1*—plays a positive role in the development of defense priming and systemic acquired resistance (SAR, Pajerowska-Mukhtar et al., 2012; Pick et al., 2012), we compared the amino acid sequences of HSFs/HSPs from *Vitis vinifera* specie and those of *Arabidopsis thaliana* HSFs by constructing a phylogenetic tree. According to the phylogenetic tree of *Vitis vinifera* HSFs/HSPs with *AtHSFs*, *VvHSP24* and *AtHSFB1* grouped into class B and a single conserved HSF_DNA-binding domain consisting of 94 amino acids is present at residue ranges of 11–104 and 6–99 in *AtHSFB1* and *VvHSP24*, respectively (Supplementary Figure 3). Hence, the expression of *VvHSP24*, an *AtHSFB1* orthologous gene in *Vitis vinifera*, might be related to grape defense. In addition, GFP-tagged *VvHSP24* was exclusively detected in the nucleus of onion epidermal cells, contrarily the positive control, i.e., GFP alone, was found in both the cytoplasm and nucleus (Figure 2). As a result, *VvHSP24* might be a nucleus-localized regulatory protein associated with the development of defense priming.

### Effect of BABA Treatment on Transcript of *VvHSP24* and SA-Dependent Genes in Grapes

**FIGURE 3:**
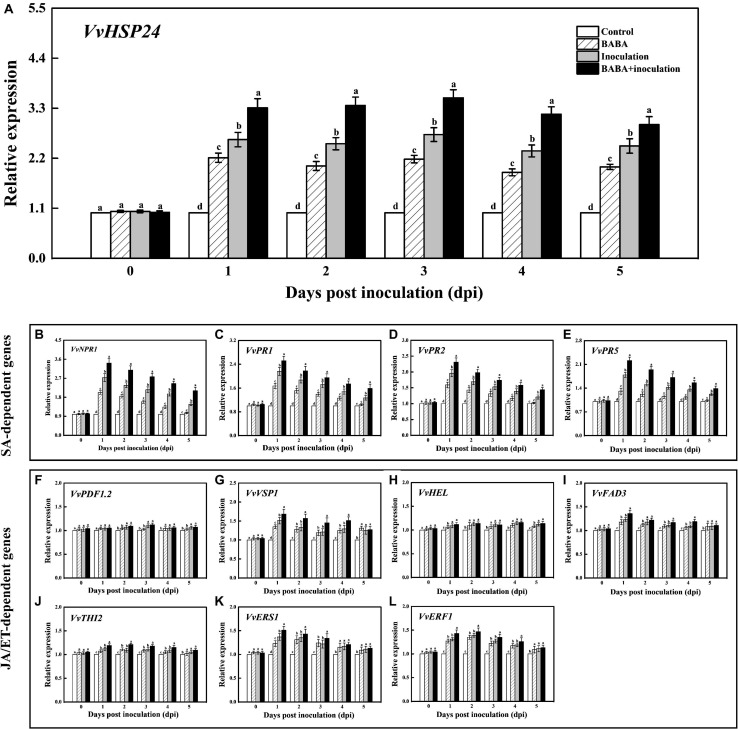
Transcript levels of *VvHSP24* **(A)**, SA-dependent *VvNPR1* **(B)**, *VvPR1* **(C)**, *VvPR2* **(D)** and *VvPR5* **(E)** genes and JA/ET-dependent *VvPDF1.2* **(F)**, *VvVSP1* **(G)**, *VvHEL* **(H)**, *VvFAD3* **(I)**, *VvTHI2* **(J)**, *VvERS1* **(K),** and *VvERF1* **(L)** genes in grapes after inoculation with *B. cinerea* for 1-day intervals. Relative mRNA expression levels are expressed as the mean ± SE. Different letters above the bar represent a significant difference between treatments (*p* = 0.05).

For analyzing the intensity of BABA-triggered SAR defense, the qRT-PCR method was used to quantify the transcript levels of the *VvHSP24* and SAR marker genes in grapes throughout 5 days of the inoculation. As shown in Figure 3A, treatment of grapes with 10 mmol L^–1^ BABA enhanced expression of the *VvHSP24* gene within the incubation period of 5 days, similar to the trend in our previous RNA-seq database^2^ (Supplementary Figure 5). Thus, *VvHSP24* may indeed participate in BABA-IR in grapes upon the perception of disease stress. In addition, although grapes reacted to BABA pretreatment with enhanced transcripts of *VvNPR1* and SA-responsive genes such as *VvPR1*, *VvPR2*, and *VvPR5* following mock inoculation with ddH_2_O, the reaction was much more pronounced in *B. cinerea*-inoculated grapes than in non-fungal-infected grapes. In BABA-treated and then *B. cinerea-*inoculated grapes, accumulation of *VvNPR1* and SA-inducible genes transiently peaked at 1 day post inoculation (dpi) followed by a gradual decrease (Figures 3B–E). Thus, SA-dependent priming of pathogen responses was boosted in BABA-pretreated grapes because the transient accumulation of mRNA transcript levels detected in SA-dependent marker genes is a hallmark of the priming mode for BABA-IR (Ton et al., 2005).

### Effect of BABA Treatment on Transcript of JA/ET-Dependent Genes in Grapes

It is well-documented that a subset of genes, including *VvPDF1.2* (encoding a plant defensin), *VvHEL* (encoding a hevein-like protein), *VvVSP1* (encoding an acid phosphatase), *VvTHI2* (Encoding a antimicrobial thionin), *VvFAD3* (encoding a fatty acid desaturase), *VvERS1* (encoding an ethylene response sensor), and *VvERF1* (encoding an ethylene response factor), are regulated by JA or ET signaling pathway and associated with a resistance against hostile threats (Zarate et al., 2007). Thus, the transcript levels of the above genes were assessed for determining the inductive effectiveness of BABA elicitation on JA/ET-dependent defense. Throughout the whole incubation period, the treatment of *B. cinerea*-inoculated grapes with 10 mmol L^–1^ BABA did not induce the significant change in the transcript levels of *VvPDF1.2*, *VvHEL*, and *VvTHI2* compared with the only *B. cinerea*-inoculated samples (Figures 3F,H,J). On the contrary, an inductive effect of BABA on gene expressions of *VvVSP1*, *VvFAD3*, *VvERS1*, and *VvERF1* could be observed in the BABA-stimulated and subsequent *B. cinerea*-challenged grapes (Figures 3G,I,K,L), indicating that the JA/ET-responsive genes were in part implicated in the BABA-induced priming defense.

### Effect of BABA Treatment on Individual Phytoalexin Content in Grapes

**FIGURE 4:**
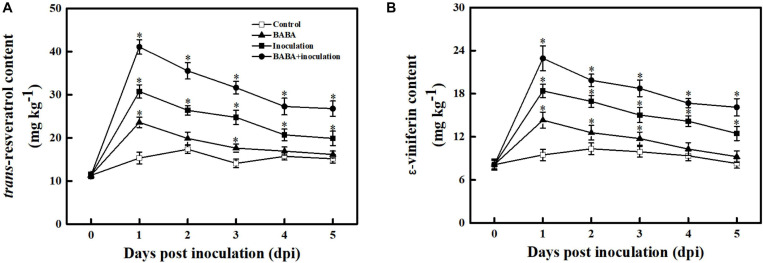
Changes in the amounts of *trans*-resveratrol **(A)** and ε-viniferin **(B)** in grapes after inoculation with *B. cinerea* at 1-day intervals. Phytoalexin contents are expressed as the mean ± SE. An asterisk represents a significant difference between pathogen- or BABA-treated and untreated grapes (*p* = 0.05).

To assess whether BABA-fueled defense was related to phytoalexin biosynthesis in grape berries, the contents of the dominant stilbenes in *Vitis vinifera* species, including *trans*-resveratrol and its oligomer ε-viniferin, were measured (Sarig et al., 1997). As depicted in Figure 4, the *trans*-resveratrol and ε-viniferin contents in the controls fluctuated within a very small range (from –23.72 to 17.06%) around their average values over 5 days at 20°C. BABA treatment alone was sufficient to promote stilbene synthesis in grapes, and subsequent *B. cinerea* inoculation resulted in an obvious enhancement of *trans*-resveratrol and ε-viniferin compared with the controls. It is worth noting that the amount of phytoalexin was higher in fruit treated with BABA elicitation in combination with *B. cinerea* suspension than that in non-*B. cinerea*-inoculated or non-BABA-treated grapes within 5 dpi, indicating the BABA elicitation exhibited a positive regulation on stilbene phytoalexin biosynthesis in grapes.

### Direct Interaction Between *VvHSP24* and *VvNPR1*

**FIGURE 5:**
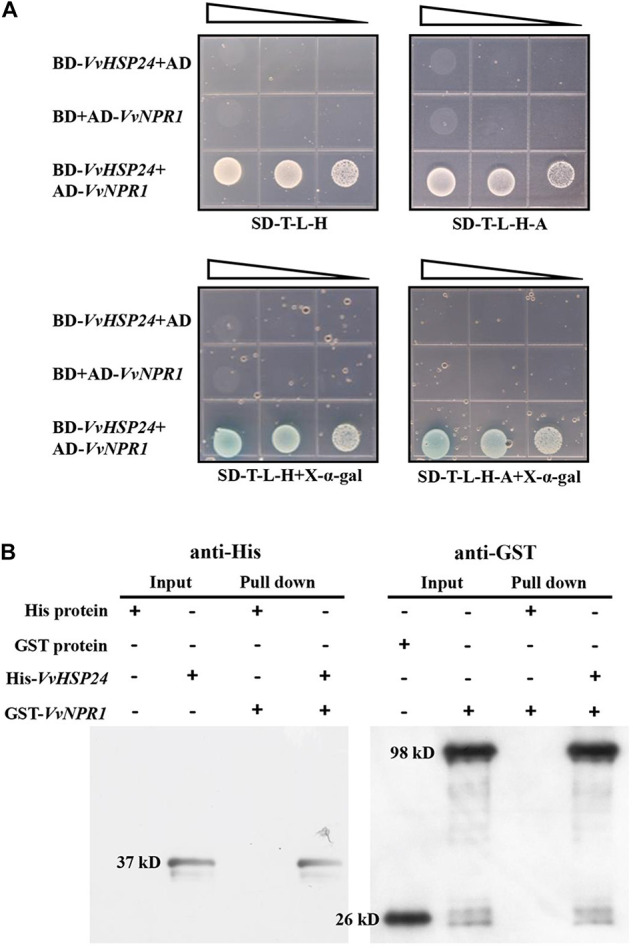
*VvHSP24* interacts with *VvNPR1 in vivo* and *in vitro*. **(A)** Yeast two-hybrid analysis of the physical interaction between *VvHSP24* and *VvNPR1*. SD-T-L-H, SD/-Trp-Leu-His agar medium; SD-T-L-H-A, SD/-Trp-Leu-His-Ade agar medium. The right-angled triangles on the top of the gridded Petri dishes represent the absorbance of yeasts at 600 nm in a 10-fold dilution series, from 1 to 10^–2^ abs. **(B)** The GST-fused *VvNPR1* protein (1 mL) was incubated with 1 mL of preimmobilized His-*VvHSP24* protein in a total volume of 25 mL at 4°C for more than 8 h. The pulled down proteins (6 μL) were analyzed by western blotting with anti-His or anti-GST antibodies.

The Y2H system was used to identify whether physical interaction occurs between *VvHSP24* and *VvNPR1 in vivo*; meanwhile, the interaction between *VvHSP24* and *VvNPR1 in vitro* was conducted by His pull-down. As shown in Figure 5A, colonies of competent yeast AH109 cotransfected with the BD-*VvHSP24* and AD-*VvNPR1* vectors grew within 4–6 days on synthetic dropout plates (SD/-Trp-Leu-His and SD/-Trp-Leu-His-Ade), and these cotransformants were blue on X-α-gal plates. Few or no colonies carrying BD-*VvHSP24* plus the empty pGADT7 vector or AD-*VvNPR1* plus the empty pGBKT7 vector were obtained. Therefore, the interaction between *VvHSP24* and *VvNPR1* was confirmed by the successful activated GAL4-responsive reporter genes.

Figure 5B illustrated that two intense bands observed at the position corresponding to the estimated sizes of His-*VvHSP24* (37 kD) or GST-*VvNPR1* (98 kD) when incubated with anti-His or anti-GST antibodies, respectively. For detection of a pulled down protein, a His-tagged *VvHSP24* fragment protein immobilized on Ni sepharose 6 Fast Flow was used as bait against the GST-*VvNPR1* fusion protein. As shown in lanes 7 and 8 of Figure 5B, GST-*VvNPR1* was pulled down by the His-*VvHSP24* fusion protein but not by the unfused His; hence, the *VvHSP24* protein physically interacted with *VvNPR1 in vitro*.

### *VvHSP24* Cannot Directly Regulate the Transcript of *PR* Genes

**FIGURE 6:**
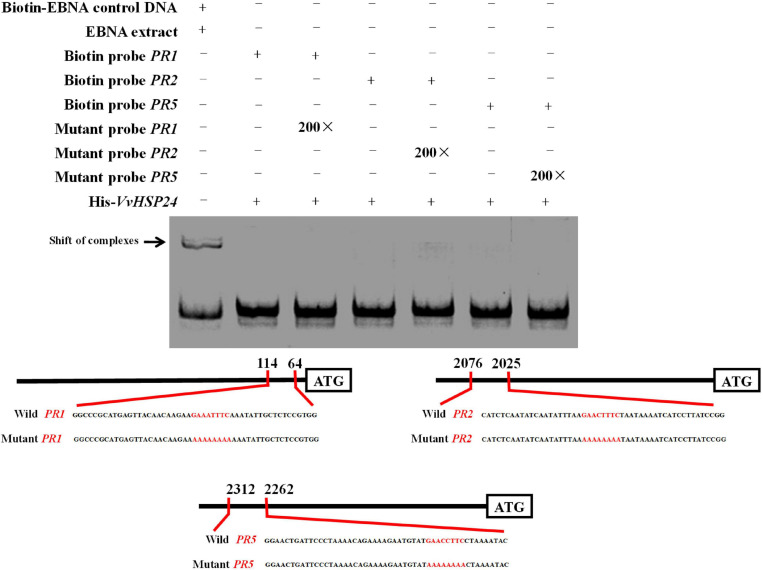
The binding patterns of *VvHSP24* to the promoters of *PR1*-, *PR2-*, and *PR5*-containing HSE motifs were detected by EMSA. The HSE elements within the probe sequences are highlighted with red letters. The recombinant His-*VvHSP24* protein was incubated with biotin-labeled probes and then the protein-DNA complexes were resolved on polyacrylamide gels and visualized by exposure of the dehydrated gels to X-ray films. The addition of 200-fold mutant probes was performed in the competitive binding assays to test for the specific of the binding assays between *VvHSP24* and *PR1*, *PR2*, and *PR5* promoters.

qRT-PCR results showed that *PR* genes such as *PR1*, *PR2*, and *PR5* display similar long-term potentiation (LTP) trends as *VvHSP24* after BABA elicitation (Figure 3). In particular, previous studies have shown that HSFs/HSPs regulate the transcript of the target genes primarily by interacting with HSF sequence-binding elements (HSEs) (Nover et al., 2001; Guertin and Lis, 2010). HSEs (5′-GAAnnTTC-3′) were detected in the promoters of all *PR* genes (Supplementary Data 1). Hence, we tested the possibility whether *VvHSP24* could directly control *PR* gene transcripts. Unexpectedly, the His-*VvHSP24* fusion protein was unable to bind the biotin-labeled probes targeting the HSE motifs from the *PR1*, *PR2*, and *PR5* promoters; indeed, mobility shifts were not observed, and the addition of mutated HSEs had no effect (Figure 6). However, a mobility shift was produced when biotin-EBNA control DNA was incubated with EBNA extract. Thus, the *VvHSP24* could not directly regulate the transcript of *PR* genes.

### Heterologous Expression of *Vitis vinifera* HSP24 Enhances the Resistance of *Arabidopsis* to *B. cinerea* and Mitigates Cell Death in Transgenic Leaves

**FIGURE 7:**
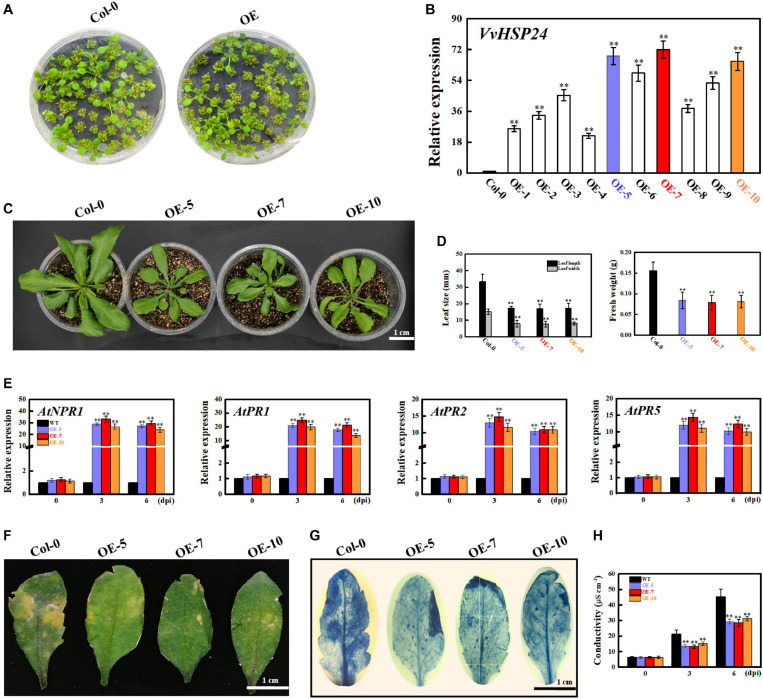
Overexpression of *VvHSP24* activates *PR* gene expression in *Arabidopsis* leaves and increases the resistance of transgenic *Arabidopsis* to the fungal pathogen *B. cinerea*. **(A)** Phenotypes of *Arabidopsis* Col-0 wildtype and transgenic seedlings for 2 weeks. **(B)** Relative mRNA levels of *VvHSP24* in Col-0 wildtype and transgenic *Arabidopsis* plants grown in soil for 4 weeks. Transcript abundances are expressed relative to the reference gene of *AtActin2* and expressed as the mean ± SE. Two asterisks indicate significant differences between wildtype and transgenic plants (*p* < 0.01). **(C,D)** Overexpression of *VvHSP24* in *Arabidopsis* inhibits growth, as exhibited by the shorter leaf length and lower biomass of the dominant *VvHSP24*-overexpressing *Arabidopsis* (OE5, OE7, and OE10) than of Col-0 wildtype. Bar = 1 cm. **(E)** qRT-PCR analysis of the transcript levels of defense-related genes (NPR1, *PR1*, *PR2*, and *PR5*) in Col-0 wildtype and *VvHSP24*-overexpressing *Arabidopsis* (OE5, OE7, and OE10) infected by *B. cinerea* at 0, 3, and 6 dpi. mRNA expression levels were quantified against the value of *AtActin2*; the data represent the mean ± SE of three separate replicates. Two asterisks indicate significant differences between wildtype and OE5, OE7, or OE10 (*p* < 0.01). **(F)** Disease symptoms of wildtype and *VvHSP24*-overexpressing (OE-5, OE-7, and OE-10) *Arabidopsis* leaves after *B. cinerea* infection at 6 dpi. Bar = 1 cm. **(G)** Necrotic areas in Col-0 wildtype and *VvHSP24*-overexpressing *Arabidopsis* leaves were determined by trypan blue staining after *B. cinerea* infection at 6 dpi, and images were captured with a digital camera. **(H)** Measurement of electrolyte leakage from wildtype and *VvHSP24*-overexpressing *Arabidopsis* leaves infected with *B. cinerea* at 6 dpi. Conductivities are expressed as the mean and SE of three independent biological replicates. Two asterisks indicate significant differences between wildtype and *VvHSP24*-overexpressing *Arabidopsis* at *p* = 0.01.

As previously reported, *AtHSFB1* is a critical player in the development of defense priming (Pajerowska-Mukhtar et al., 2012; Pick et al., 2012). To examine the effectiveness of *VvHSP24* (an orthologous gene of *AtHSFB1* from *Vitis vinifera*) in necrotrophic pathogen-induced defense responses, transcript levels of *VvHSP24* and pathogenesis-related genes (NPR1, *PR1*, *PR2*, and *PR5*) in *B. cinerea*-inoculated WT plants and *VvHSP24-*overexpressing lines were monitored. qRT-PCR analysis showed that *VvHSP24* gene expression was significantly enhanced in the *VvHSP24*-overexpressing lines, especially in OE-5, OE-7, and OE-10, in comparison with Col-0 WT (Figures 7A,B). As shown in Figure 7E, mRNA levels of *AtNPR1*, *AtPR1*, *AtPR2*, and *AtPR5* were upregulated in the three *VvHSP24*-overexpressing lines (OE-5, OE-7, and OE-10) at 3 and 6 dpi compared with WT plants. To determine pathogen growth- and cell death-inducing activity, we further observed *B. cinerea*-inoculated leaves from WT and *VvHSP24*-transgenic plants by imaging after staining with lactophenol trypan blue. At 6 days after *B. cinerea* inoculation, almost all of the cells of WT leaves were basically dead and stained with trypan blue; in contrast, lesions and necrotic areas were visibly reduced in all transgenic lines with respect to dot-inoculated WT plants (Figures 7F,G). Coincidently, cell electrolyte leakage from transgenic leaves infiltrated with the *B. cinerea* conidial suspension was also significantly lower than that from WT plants (Figure 7H). Of note, heterologous expression of HSP24 from *Vitis vinifera* apparently retarded the growth of transgenic *Arabidopsis* plants, as displayed with dwarf leaves, smaller leaf area and less biomass in the transgenic lines than in the WT plants (Figures 7C,D). These data reflected that overexpression of *VvHSP24* can obviously alleviate cell damage and disease progression.

### *AtHSFB1* Mutants With Decreased Resistance in Response to Fungal Pathogen

**FIGURE 8:**
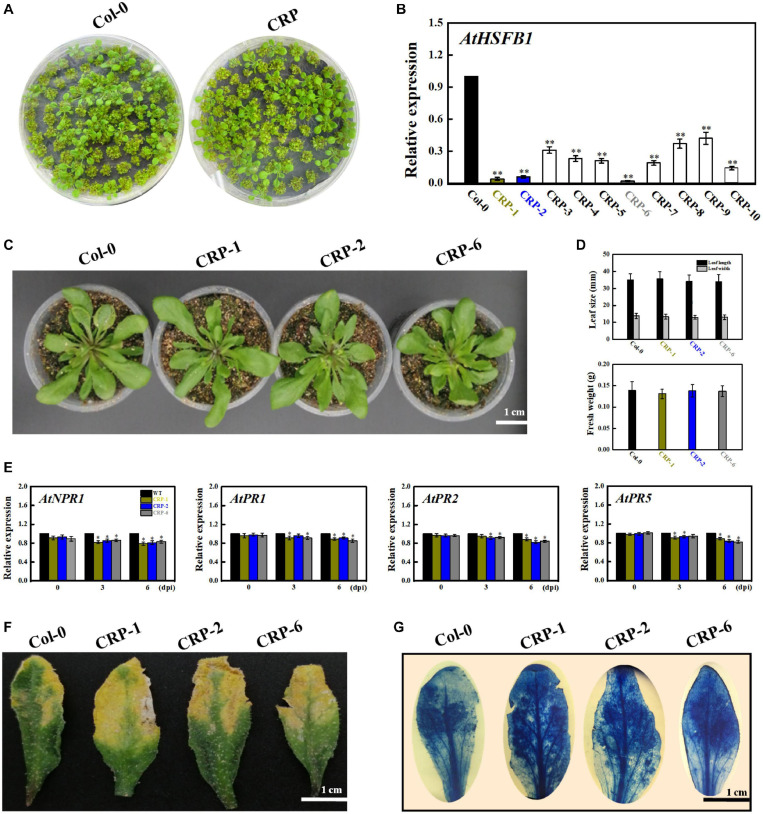
HSFB1 mutation mediated by the CRISPR/Cas9 system scarcely modifies the fungal resistance of *Arabidopsis* to *B. cinerea*. **(A)** Growth phenotypes of *Arabidopsis* Col-0 wildtype and mutant seedlings at 15 days after sowing on MS plants. **(B)** Transcript levels of HSFB1 from Col-0 wildtype and CRISPR mutants grown in soil for more than 4 weeks. Transcript amounts are normalized against the *AtActin2* value, and the results are expressed as the mean ± SE. Asterisks indicate that the differences between wildtype and CRISPR mutants are statistically extremely significant at the *p* = 0.01 level. **(C,D)** CRISPR mutants (CRP1, CRP2, and CRP6) show unchanged levels of leaf length and biomass compared with Col-0 wildtype. Bar = 1 cm. **(E)** Relative mRNA levels of NPR1, *PR1*, *PR2*, and *PR5* genes in Col-0 wildtype and CRISPR mutants (CRP1, CRP2, and CRP6) after inoculation with *B. cinerea* at 0, 3, and 6 days. Asterisks represent significant differences between wildtype and CRP1, CRP2, or CRP6 at *p* = 0.01. **(F)** Disease symptoms of Col-0 wildtype and CRISPR mutants (CRP1, CRP2, and CRP6) at 6 dpi. Bar = 1 cm. **(G)** Necrotic areas in wildtype and CRISPR mutants were stained with trypan blue and recorded by a camera mounted on a binocular stereomicroscope.

Given that *AtHSFB1* is a *VvHSP24* orthologous gene in *Arabidopsis*, we harvested *Arabidopsis AtHSFB1* mutant seedlings constructed via the CRISPR-Cas9 system to evaluate the disease resistance of HSP through mutant-based verification. As depicted in Figures 8A,B, *AtHSFB1* transcript in all CRISPR mutants, especially in CRP-1, CRP-2, and CRP-6, were largely not expressed or drastically decreased compared with that in WT plants. Interestingly, the dominant CRISPR mutants CRP-1, CRP-2, and CRP-6 showed no apparent alteration in growth phenotypes compared with those in the WT (Figures 8C,D). In addition, CRISPR/Cas9-mediated *AtHSFB1* knockout lines displayed similar kinetics and intensity of pathogenesis-related gene (NPR1, *PR1*, *PR2*, and *PR5*) transcript accumulations, and all were slightly or significantly decreased when compared with the WT at 3 and 6 dpi (Figure 8E). Consistent with this observation, the symptoms of fungal infection and staining level of the dot-inoculated mutants were stronger than those of WT leaves (Figures 8F,G). Thus, the *AtHSFB1* mutation impaired the defense expression and led to a pathogenic sensitiveness.

## Discussion

As sessile organisms, plants are frequently subjected to a fluctuating environment and exhibit a high degree of elasticity in survival under various stresses, suggesting broad crosstalk between plant defense responses and other biochemical and physiological processes (Khare et al., 2020). Of note, hyperactivation of resistance not only cannot terminate the wide variety of disease threats but also may entail an irreversible cost that might suppress the growth, fruit set, yield and quality of horticultural crops (Vos et al., 2013). Priming has been found to be an ecological approach of resistance owing to its capacity to respond promptly to a certain degree of biotic stress; thus, priming can balance the specific resistance and the resulting fitness costs or quality impairment in crops (Conrath et al., 2015; Ton et al., 2009). The induction of BABA on biotic resistance has been researching over half a century (Cohen et al., 2016). In this study, the resistance against *B. cinerea* fungal infection of postharvest grapes elicited by BABA at 10 mmol L^–1^ likely paralleled a priming mode. In fact, its definite role in the transient activation antimicrobial protein expression, particularly pathogenesis-related proteins (including *VvNPR1*, *VvPR1*, *VvPR2*, and *VvPR5*), and the synergistic stimulation of host-synthesized antifungal compounds such as *trans*-resveratrol and ε-viniferin occurred exclusively after combined therapy (BABA pretreatment with fungal infection; Figures 1, 3, 4; Thevenet et al., 2017). Although the infection process of the gray mold fungus *B. cinerea* could not be completely abolished by 10 mmol L^–1^ BABA, disease development was appreciably attenuated, leading to the smaller lesions and lower disease incidences during 5 days of the incubation at 20°C (Figure 1 and Supplementary Figure 1). Similarly, such priming resistance against pathogen stresses in *Arabidopsis*, peach and bayberry has been observed for other priming elicitors, such as methyl jasmonate (MeJA), 2,4-epibrassinolide (EBR), benzothiadiazole (BTH), and chitosan (Beckers et al., 2009; Wang et al., 2014; De Vega et al., 2020; Li et al., 2020a,b). In addition, Wang et al. (2016) reported an *in vitro* lethal activity of BABA on the cell membrane of *B. cinerea*. Thus, the combination of the enhanced host disease resistance elicited by BABA and its direct toxic effect on pathogens supports the inherent role of BABA as a defense inducer. Although the BABA conferred a positive resistance on disease development caused by *B. cinerea*, the combination strategy of BABA and other chemical or physical inducers should be tested for gaining a more satisfactory protection from fungal attack.

Specifically, primed grapes presented much stronger transcript levels of SA-responsive defense-related genes, such as *VvPR1*, *VvPR2*, and *VvPR5*, and stimulated disease resistance upon the artificial *B. cinerea*-inoculation over the observation period. We speculate that the mode of manner of BABA-elicited defense in grapes may be attributed to a SAR-like defense response. Although Nie et al. (2017) have recently pointed out that JA/ET-dependent signaling pathways have been shown to be involved in systemic resistance against *B. cinerea* in *Arabidopsis*, a set of JA/ET-dependent defensive genes (*VvPDF1.2*, *VvHEL*, and *VvTHI2*) were not promoted in *B. cinerea*-inoculated grapes after the BABA elicitation in our present study. In contrast, the transcriptional data exhibited a determinable induction of BABA on gene expressions of *VvVSP1*, *VvFAD3*, *VvERS1*, and *VvERF1* in grapes. Noticeably, either of *VvPDF1.2*, *VvTHI2*, or *VvHEL* can directly participate in defense expression against biotic stress (Oh et al., 1999; Bertini et al., 2012), but the other JA/ET-dependent genes including *VvVSP1*, *VvFAD3*, *VvERS1*, and *VvERF1* have been reported to be relevant to the plant development, ethylene perception, metabolic process or mechanical wounding healing, rather than the direct pathogenic response (Nath and Watson, 1980; Puttick et al., 2009; Liu and Wen, 2012). Hence, this BABA-IR in grapes is mainly associated with the expression of SA-dependent SAR. These results were in part consistent with our most current findings, showing that the BABA-activated resistance in grapes and peaches is dependent on SA signaling but not the JA/ET signaling pathways (Li et al., 2020c; Wang et al., 2021). Takahashi et al. (2004), Koornneef et al. (2008), and van der Does et al. (2013) also elucidated that the SA-dependent SAR reaction suppressed the transduction of JA signaling and the corresponding expression levels of JA-dependent defensive genes in model plant *Arabidopsis thaliana*. However, the obvious increases in the transcript levels of *VvVSP1*, *VvFAD3*, *VvERS1*, and *VvERF1* in BABA-treated grapes provided a valuable clue that BABA may affect the fruit physiology and quality due to the genetic regulation of metabolic flow between the fruit defense and development, which will lead us to research the relationship between the induced resistance and its allocated metabolic influence. Coincidentally, the protection of *Arabidopsis* and tobacco plants by BABA against infection by the necrotrophic fungus *B. cinerea*, virulent pathogen tobacco mosaic virus (TMV) or avirulent pathogen *Pseudomonas syringae* pv. *tomato* (*Pst*) through the burst of the SA-dependent SAR pathway has been reported (Siegrist et al., 2000; Zimmerli et al., 2001; Slaughter et al., 2012). These results suggest that BABA-IR might be dependent on the SA-dependent defense pathway and several potential factors may propagate the SA signal.

Heat shock proteins are members of multigene families encoding molecular chaperones, and have long been recognized as heat stress-inducible proteins. However, a large number of evidence have pointed out that HSPs become activated when plants undergo other stresses, including drought, salt, cold, heavy metals, oxidative stresses and pathogen invasion, demonstrating extensive transcriptional overlap among heat and non-heat stress response pathways, and the essential role of HSPs/chaperones in the crosstalk of multistress resistance (Swindell et al., 2007; Jacob et al., 2017). A report by Pick et al. (2012) indicated that HSFB1, a class B heat shock factor, promotes primed expression of a subset of defense-related genes and is a potential candidate for SAR in *Arabidopsis* against *Pseudomonas syringae*. Windram et al. (2012) also implied a crosstalk mediating heat stress and *B. cinerea* infection. Similarly, a series of notable transcriptomic analysis by Sham et al. (2014, 2015, 2017) have emphasized that HSPs shared signaling pathways responding to biotic and abiotic stresses in *Arabidopsis*. In those cases, the specific HSP17.4 or HSP26.5-P was up- or down-regulated by the *B. cinerea* infection respectively, indicating that HSPs may be implicated in defense function. Hence, to explore whether a credible heat shock factor that also participates in the development of defense priming and SAR in grape berries might exist, we first comparatively analyzed the phylogenetic relationship between *Vitis vinifera* HSFs/HSPs and *Arabidopsis thaliana* HSFs (*AtHSFs*). A HSP (*VvHSP24*) with close sequence similarity to *AtHSFB1* from *Arabidopsis* was identified and isolated (Supplementary Figure 3). Multiple alignment and conserved domain analysis of *VvHSP24* and *AtHSFB1* further revealed that both factors contain a highly conserved HSF_DNA-binding domain consisting of a total of 94 amino acids. Furthermore, *VvHSP24* and *AtHSFB1* group into class B, indicating that *VvHSP24*, as an ortholog of *AtHSFB1*, might exert a certain effect on SAR in grapes against the necrotrophic fungus *B. cinerea* (Supplementary Figure 3). Indeed, we found that treatment of grapes with a formulation of BABA increased the expression of *VvHSP24* mRNA by several-fold, and the transcript level of *VvHSP24* was significantly activated by *B. cinerea* fungal infection (Figure 3A). Together, these observations support our assumption that *VvHSP24* is a candidate for BABA-induced systemic resistance in grape berries. Intriguingly, most *npr1* (for non-expresser *PR* genes) alleles and NPR1 paralog null mutants exhibit enhanced disease susceptibility phenotype and are incapable of expressing *PR* genes (including *PR1*, *PR2*, and *PR5*) or developing SAR in their response to SA or its analog INA (2,6-dichloroisonicotinic acid) and benzothiadiazole (BTH); this reveals the positive function of NPR1 in SA-mediated activation of *PR* genes and SAR development (Canet et al., 2010). Because the dominant transgenic *Arabidopsis* plants overexpressing *VvHSP24* exhibited potentiated resistance to necrotrophic fungal pathogens, including dozen-fold increased expression of *VvNPR1* and the resulting constitutive expression of SA-responsive *PR1*, *PR2*, and *PR5* genes and decreased levels in necrotic areas and cell death (Figures 7E–H), it can be deduced that *VvHSP24* functions in the defense signaling pathway upstream of *VvNPR1*. This scenario would require the generation of the corresponding *VvHSP24* mutation for confirmation. However, constitutive expression of NPR1 and SA-responsive genes in the CRISPR mutants of *AtHSFB1* (an orthologous gene of *VvHSP24* in *Arabidopsis*) was only slightly suppressed compared with that in the WT (Figure 8E). This finding elucidated that the NPR1-conferred resistance phenotype does not depend on HSP/chaperone; thus, HSP/chaperone is not a direct upstream regulator of NPR1.

If HSP/chaperone is not an upstream regulator of NPR1, where does it function in the defense signaling pathway? The presence of ankyrin repeats but the lack of a DNA-binding domain indicate that NPR1 may execute its regulatory role in SAR through interaction with other proteins (Dong et al., 2001). Therefore, it is possible that HSP24 physically interacts with NPR1 to stimulate the development of SAR against *B. cinerea* fungal infection in grape berries. As depicted in Figures 2, 5, *VvHSP24* was identified as a nuclear chaperone that interacted directly with the *VvNPR1* in both *in vivo* and *in vitro* systems, as expected. Additionally, the results of EMSA (Figure 6) show that *VvHSP24* did not have the capacity of activating the transcript of the *PR* genes directly, implying that the protein binding activity of *VvHSP24* to *VvNPR1* is a prerequisite for *PR* expressions and consequent defense functions. In contrast to the resistance of transgenic *Arabidopsis* plants overexpressing *VvHSP24* against fungal pathogens over 6 dpi, SA-responsive *PR* gene expressions and resistance to the necrotrophic fungus *B. cinerea* in the CRISPR mutants CRP1, CRP2 and CRP6 were not constitutively repressed. Moreover, an interesting observation of our study is the finding that unlike the limited influence of CRISPR mutation on transgenic plants, the high production of *VvHSP24* in transgenic *Arabidopsis* overexpressing this gene can be beneficial for enhancing plant fungal resistance but can cause deleterious effects on plant growth (Figures 7C,D, 8C,D). Such negative influence of *VvHSP24* overexpression on biomass and leaf length may occur because *VvHSP24* initiates specific transcriptional reprogramming that prioritizes defense over growth-related costs. In fact, Pajerowska-Mukhtar et al. (2012) found that *AtHSFB1*, the *VvHSP24* orthologous gene in *Arabidopsis*, plays a major role in the growth-toward-defense transition. Although overexpressing the stress protein HSP24 distinctly elevated the expression levels of *NPR1* and SA-inducible genes, the CRISPR mutants only presented a slight decrease in NPR1, *PR1*, *PR2*, and *PR5* transcript levels. Conversely, a more persuasive hypothesis is that HSPs/chaperones might not separately affect the defense signaling pathway downstream of *VvNPR1*, leading to *PR* gene expressions and resistance phenotypes. One probable explanation is that a second protein or factor, in addition to HSP/chaperone, is required for NPR1 activation in the defense signaling pathway. The CRISPR mutants might not totally bypass the positive function of this second protein or factor. In support of this possibility, Yu et al. (2001), Desveaux et al. (2002), Després et al. (2003), and Dong (2004) demonstrated that in addition to TGAs, the single-stranded DNA-binding protein Whirly1 and WRKY transcription factors are involved in the NPR1-mediated SAR network. Similarly, our previous studies showed that the involvement of TGA1 and WRKY transcription factors in the activation of fungal resistance progresses in peaches and grapes in an NPR1-dependent manner (Li et al., 2020c,d; Wang et al., 2020). Indeed, the NPR1 protein tends to form a large-scale oligomer under natural conditions, which seriously impedes its cellular functions (Tada et al., 2008), and the main chaperone function of HSPs is to modulate the depolymerization of bound substrates (Wang et al., 2004; Haslbeck et al., 2015). Thus, the stress protein HSP24 might control an important switch that, depending on fungal stress, may differentially disaggregate NPR1 into an active monomer to regulate NPR1-dependent SA signaling pathway. Similarly, Ramakrishna et al. (2003) found that the HSP viscosity 1 (*visl*) acts a positive function in pectin depolymerization. Moreover, BABA-IR, as exhibited in our and other studies, is a crosstalk mechanism through a convergence of several signaling pathways, such as the SA, ABA, PI, JA, and ET pathways, further suggesting the existence of shared components other than NPR1, TGAs, WRKYs and Whirly among these defense pathways. In terms of the SA signaling pathway, our current results suggest that BABA-IR in grapes can be fueled by the *VvNPR1*-*VvHSP24* complex when *VvNPR1* oligomers become disaggregated, resulting in producing active monomers that propagate the SA signal and induce *PR* gene expressions.

## Conclusion

We characterized a HSP, *VvHSP24*, as a potential candidate in NPR1-dependent plant resistance to the necrotrophic fungus *B. cinerea*. Furthermore, NPR1 is an essential regulator in the development of SAR and a key regulatory protein in activating transcripts of SA-inducible genes, but this capacity of *VvNPR1* might rely on posttranslational modification of the *VvHSP24* chaperone and their subsequent interaction after the perception of invading disease stress by plant cells. Our study reports the previously unclarified function of HSPs/chaperones and offers insight for uncovering the regulatory network of disease resistance in agronomic fruits. Given that the HSPs usually generate upon the plant suffers from various stresses, further researches should concentrate on the elaboration of the explicit molecular or metabolic modes of the cross-tolerance in postharvest fruit. Meanwhile, identification of stress-related HSPs and their functional co-activator under biotic stress may be definitely conducive to the studies on induced resistance.

## Data Availability Statement

The original contributions presented in the study are included in the article/Supplementary Material, further inquiries can be directed to the corresponding author/s.

## Author Contributions

KW, SC, YZ, and FX conceived the experiments. CLi, SC, KW, CLe, NJ, YJ, and LQ conducted the experiments. CLi and KW analyzed the data and wrote the manuscript. All the authors read, commented, revised, and approved the final revision manuscript.

## Conflict of Interest

The authors declare that the research was conducted in the absence of any commercial or financial relationships that could be construed as a potential conflict of interest.

## Footnotes

1
https://crispr.dbcls.jp/

2
https://www.ncbi.nlm.nih.gov/sra/PRJNA663112

## Supplementary Material

Supplementary Figure 1 Tree analysis of the isolated *Botrytis cinerea* strain with the *Botrytis cinerea* family. Accession numbers of sequences: *Botrytis cinerea* B05.10, MH427722.1; *Botryotinia fuckeliana* T4, AJ422103.1; *Botrytis cinerea* Bo.CC166, MH427722.1; *Botrytis cinerea* strain B23, AJ422103.1; *Botrytis cinerea* BOT 40, KU145363.1; *Botrytis cinerea* BOT 5, KX772771.1; *Botrytis cinerea* BOT 16, KX781162.1; *Botrytis cinerea* BOT 61, KX781165.1; *Botryotinia fuckeliana* strain Minhang, AY694146.1; *Botrytis cinerea* BOT 74, KX781167.1; *Botrytis cinerea* strain SCB7-5, KR080285.1; *Botrytis cinerea* Bot 68, KU145391.1; *Botrytis cinerea* strain SCB2-2, KR080283.1; *Botrytis cinerea* strain SCB7-4, KP165498.1; *Botrytis cinerea* strain SCB5-2, KP165495.1; *Botrytis cinerea* strain BC-8, KP141792.1; *Botryotinia fuckeliana* species; AY674786.1; *Botrytis cinerea* strain KBC-13, KP141795.1; *Botrytis cinerea* strain SL, KU936083.1; *Botrytis cinerea* strain BC-10, KP141793.1; *Botrytis cinerea* strain KBC-14, KP141794.1; *Botrytis cinerea* strain BC-1, KP141790.1; *Botrytis cinerea* strain AC1, KU936079.1; *Botrytis cinerea* strain AR, KU936082.1; *Botrytis cinerea* strain SV, KU936085.1.

Supplementary Figure 2 Grape fruit displays significant differences in visual appearances under four treatments (control, BABA, inoculation, and BABA + inoculation). BABA treatment and pathogen inoculation were conducted independently twice, with similar results. Representative grapes were photographed and compared after the 5 dpi at 20°C.

Supplementary Figure 3 Phylogenetic and domain analyses of *VvHSPs*/*AtHSFs*. **(A)** Three classes of HSFs/HSPs are shown in the phylogenetic tree. *VvHSP24* and *AtHSFB1* are in bold in purple and group into class B. The sequences of 57 HSFs/HSPs from *Vitis vinifera* and *Arabidopsis thaliana* were aligned with Clustal W, and the phylogenetic analysis was made with MEGA (NJ method; bootstrap of 1000). **(B)** Identical amino acids between *VvHSP24* and its closest related protein *AtHSFB1* are shaded in black. In addition, a single conserved HSF_DNA-binding domain consisting of a total of 94 amino acids was visualized by TBtools software; it occurs at residue ranges of amino acids 6–99 in *VvHSP24* and 11–104 in *AtHSFB1*.

Supplementary Figure 4 Schematic diagram of the M2CRISPR/Cas9-*AtHSFB1* vector.

Supplementary Figure 5 Heat map of *VvHSPs* in pairwise comparisons of BABA-vs.-control, inoculation-vs.-control, BABA + inoculation-vs.-control, and BABA + inoculation-vs.-inoculation.

Supplementary Figure 6 His-tagged protein was detected by a gel image analysis (un-cropped gels).

Supplementary Figure 7 In the His pull-down assay, GST-*VvNPR1* fusion protein was efficiently pull-downed by His-*VvHSP24* fusion protein (un-cropped gels).

Supplementary Figure 8 EMSA of *VvHSP24* binding to the HSE elements of *PR1*, *PR2*, and *PR5* (un-cropped blots).

Supplementary Table 1 Primer sequence of the used genes.

Supplementary Data 1 Promoter sequences of *VvPR1*, *VvPR2*, and *VvPR5*. HSEs (GAAnnTTC) and the translation start site (ATG) are marked in red and yellow boxed, respectively.

## References

[B1] AbuQamar S. Moustafa K. Tran L. S. (2017). Mechanisms and strategies of plant defense against *Botrytis cinerea*. *Crit. Rev. Biotechnol.* 37 262–274. 10.1080/07388551.2016.1271767 28056558

[B2] Baccelli I. Glauser G. Mauch-Mani B. (2017). The accumulation of β-aminobutyric acid is controlled by the plant’s immune system. *Planta* 246 791–796. 10.1007/s00425-017-2751-3 28762076

[B3] Balic I. Ejsmentewicz T. Sanhueza D. Silva C. Peredo T. Olmedo P. (2014). Biochemical and physiological study of the firmness of table grape berries. *Postharvest Biol. Technol.* 93 15–23. 10.1016/j.postharvbio.2014.02.001

[B4] Baniwal S. K. Bharti K. Chan K. Y. Fauth M. Ganguli A. Kotak S. (2004). Heat stress response in plants: a complex game with chaperones and more than twenty heat stress transcription factors. *J. Biosci.* 29 471–487. 10.1007/BF02712120 15625403

[B5] Beckers G. J. Jaskiewicz M. Liu Y. Underwood W. R. He S. Y. Zhang S. (2009). Mitogen-activated protein kinases 3 and 6 are required for full priming of stress responses in *Arabidopsis thaliana*. *Plant Cell* 21 944–953. 10.1105/tpc.108.062158 19318610 PMC2671697

[B6] Bertini L. Proietti S. Aleandri M. P. Mondello F. Sandini S. Caporale C. (2012). Modular structure of HEL protein from *Arabidopsis* reveals new potential functions for PR-4 proteins. *Biol. Chem.* 1 1–14. 10.1515/hsz-2012-0225 22868784

[B7] Bharti K. Schmidt E. Lyck R. Heerklotz D. Bublak D. Scharf K. D. (2000). Isolation and characterization of HsfA3, a new heat stress transcription factor of *Lycopersicon peruvianum*. *Plant J.* 22 355–365. 10.1046/j.1365-313x.2000.00746.x 10849352

[B8] Burketova L. Trda L. Ott P. G. Valentova O. (2015). Bio-based resistance inducers for sustainable plant protection against pathogens. *Biotechnol. Adv.* 33 994–1004. 10.1016/j.biotechadv.2015.01.004 25617476

[B9] Cai J. H. Chen T. Wang Y. Qin G. Z. Tian S. P. (2020). SlREM1 triggers cell death by activating an oxidative burst and other regulators. *Plant Physiol.* 183 717–732. 10.1104/pp.20.00120 32317359 PMC7271787

[B10] Canet J. V. Dobón A. Roig A. Tornero P. (2010). Structure-function analysis of npr1 alleles in *Arabidopsis* reveals a role for its paralogs in the perception of salicylic acid. *Plant Cell Environ.* 33 1911–1922. 10.1111/j.1365-3040.2010.02194.x 20561252

[B11] Chen C. Chen H. Zhang Y. Thomas H. R. Frank M. H. He Y. (2020). TBtools: an integrative toolkit developed for interactive analyses of big biological data. *Mol. Plant.* 13 1194–1202. 10.1016/j.molp.2020.06.009 32585190

[B12] Clough S. J. Bent A. F. (1998). Floral dip: a simplified method for *Agrobacterium*-mediated transformation of *Arabidopsis thaliana*. *Plant J.* 16 735–743. 10.1046/j.1365-313x.1998.00343.x 10069079

[B13] Cohen Y. Vaknin M. Mauch-Mani B. (2016). BABA-induced resistance: milestones along a 55-year journey. *Phytoparasitica* 44 513–538. 10.1007/s12600-016-0546-x

[B14] Conrath U. Beckers G. J. M. Langenbach C. J. G. Jaskiewicz M. R. (2015). Priming for enhanced defense. *Annu. Rev. Phytopathol.* 53 97–119. 10.1146/annurev-phyto-080614-120132 26070330

[B15] Dai Z. W. Ollat N. Gomes E. Decroocq S. Tandonnet J. P. Bordenave L. (2011). Ecophysiological, genetic, and molecular causes of variation in grape berry weight and composition: a review. *Am. J. Enol. Vitic.* 62 413–425. 10.5344/ajev.2011.10116

[B16] De Vega D. Holden N. Hedley P. E. Morris J. Luna E. Newton A. (2020). Chitosan primes plant defence mechanisms against *Botrytis cinerea*, including expression of Avr9/Cf-9 rapidly elicited genes. *Plant Cell Environ.* 44 290–303. 10.1111/pce.13921 33094513 PMC7821246

[B17] Després C. Chubak C. Rochon A. Clark R. Bethune T. Desveaux D. (2003). The *Arabidopsis* NPR1 disease resistance protein is a novel cofactor that confers redox regulation of DNA binding activity to the basic domain/leucine zipper transcription factor TGA1. *Plant Cell* 15 2181–2191. 10.1105/tpc.012849 12953119 PMC181339

[B18] Desveaux D. Allard J. Brisson N. Sygusch J. (2002). A new family of plant transcription factors displays a novel ssDNA-binding surface. *Nat. Struct. Biol.* 9 512–517. 10.1038/nsb814 12080340

[B19] Dong X. N. (2004). NPR1, all things considered. *Curr. Opin. Plant Biol.* 7 547–552. 10.1016/j.pbi.2004.07.005 15337097

[B20] Dong X. N. Li X. Zhang Y. L. Fan W. H. Kinkema M. Clarke J. (2001). Regulation of systemic acquired resistance by NPR1 and its partners. *Novartis. Found. Symp.* 236 165–173. 10.1002/9780470515778.ch12 11387978

[B21] Feliziani E. Smilanick J. L. Margosan D. A. Mansour M. F. Romanazzi G. Gu S. (2013). Preharvest fungicide potassium sorbate, or chitosan use on quality and storage decay of table grapes. *Plant Dis.* 97 307–314. 10.1094/PDIS-12-11-1043-RE 30722398

[B22] Gabler F. M. Smilanick J. L. Mansour M. Ramming D. W. Mackey B. E. (2003). Correlations of morphological, anatomical and chemical features of grape berries with resistance to *Botrytis cinerea*. *Phytopathology* 93 1263–1273. 10.1094/PHYTO.2003.93.10.1263 18944326

[B23] Guertin M. J. Lis J. T. (2010). Chromatin landscape dictates HSF binding to target DNA elements. *PLoS Genet.* 6:e1001114. 10.1371/journal.pgen.1001114 20844575 PMC2936546

[B24] Guo M. Liu J. H. Ma X. Luo D. X. Gong Z. H. Lu M. H. (2016). The plant heat stress transcription factors (HSFs): structure, regulation, and function in response to abiotic stresses. *Front. Plant Sci.* 7:114. 10.3389/fpls.2016.00114 26904076 PMC4746267

[B25] Haslbeck M. Weinkauf S. Buchner J. (2015). “Regulation of the chaperone function of small Hsps,” in *The Big Book on Small Heat Shock Proteins. Heat Shock Proteins*, Vol. 8 eds Tanguay R. Hightower L. (Cham: Springer), 10.1007/978-3-319-16077-1_6

[B26] Jacob P. Hirt H. Bendahmane A. (2017). The heat-shock protein/chaperone network and multiple stress resistance. *Plant Biotechnol. J.* 15 405–414. 10.1111/pbi.12659 27860233 PMC5362687

[B27] Khare S. Singh N. B. Singh A. Hussain I. Niharika K. Yadav V. (2020). Plant secondary metabolites synthesis and their regulations under biotic and abiotic constraints. *J. Plant Biol.* 11 1–14. 10.1007/s12374-020-09245-7

[B28] Koornneef A. Leon-Reyes A. Ritsema T. Verhage A. Den Otter F. C. van Loon L. C. (2008). Kinetics of salicylate-mediated suppression of jasmonate signaling reveal a role for redox modulation. *Plant Physiol.* 147 1358–1368. 10.1104/pp.108.121392 18539774 PMC2442557

[B29] Larkindale J. Vierling E. (2008). Core genome responses involved in acclimation to high temperature. *Plant Physiol.* 146 748–761. 10.1104/pp.107.112060 18055584 PMC2245833

[B30] Letunic I. Bork P. (2018). 20 Years of the SMART protein domain annotation resource. *Nucleic Acids Res.* 46 D493–D496. 10.1093/nar/gkx922 29040681 PMC5753352

[B31] Li C. H. Du M. Y. Wang K. T. (2020a). 2,4-Epibrassionolide activates priming resistance against *Rhizopus stolonifer* infection in peach fruit. *Acta. Aliment.* 49 135–143. 10.1556/066.2020.49.2.2

[B32] Li C. H. Wang J. Ji N. N. Lei C. Y. Zhou D. X. Zheng Y. H. (2020b). PpHOS1, a RING E3 ubiquitin ligase, interacts with PpWRKY22 in the BABA-induced priming defense of peach fruit against *Rhizopus stolonifer*. *Postharvest Biol. Technol.* 159:111029. 10.1016/j.postharvbio.2019.111029

[B33] Li C. H. Wang K. T. Lei C. Y. Zheng Y. H. (2020c). Translocation of PpNPR1 is required for β-aminobutyric acid-triggered resistance against *Rhizopus stolonifer* in peach fruit. *Sci. Hortic.* 272:109556. 10.1016/j.scienta.2020.109556

[B34] Li C. H. Wang K. T. Zheng Y. H. (2020d). Redox status regulates subcelluar localization of PpTGA1 associated with a BABA-induced priming defence against *Rhizopus stolonifer*. *Mol. Biol. Rep.* 47 6657–6668. 10.1007/s11033-020-05719-6 32794133

[B35] Li J. Soroka J. Buchner J. (2012). The Hsp90 chaperone machinery: conformational dynamics and regulation by cochaperones. *Biochim. Biophys. Acta.* 3 624–635. 10.1016/j.bbamcr.2011.09.003 21951723

[B36] Li R. Liu C. X. Zhao R. R. Wang L. Chen L. Yu W. Q. (2019). CRISPR/Cas9-mediated SlNPR1 mutagenesis reduces tomato plant drought tolerance. *BMC Plant Biol.* 19:38. 10.1186/s12870-018-1627-4 30669982 PMC6341727

[B37] Liu Q. Wen C. K. (2012). *Arabidopsis* ETR1 and ERS1 differentially repress the ethylene response in combination with other ethylene receptor genes1[w]. *Plant Physiol.* 158 1193–1207. 10.1104/pp.111.187757 22227969 PMC3291259

[B38] Livak K. J. Schmittgen T. D. (2001). Analysis of relative gene expression data using real-time quantitative PCR and the 2-ΔΔCT method. *Methods* 25 402–408. 10.1006/meth.2001.1262 11846609

[B39] Lovato A. Zenoni S. Tornielli G. B. Colombo T. Vandelle E. Polverari A. (2019). Specific molecular interactions between *Vitis vinifera* and *Botrytis cinerea* are required for noble rot development in grape berries. *Postharvest Biol. Technol.* 156 110924–110938. 10.1016/j.postharvbio.2019.05.025

[B40] Lowder L. G. Zhang D. W. Baltes N. J. Paul J. W. III Tang X. Zheng X. L. (2015). A CRISPR/Cas9 toolbox for multiplexed plant genome editing and transcriptional regulation. *Plant Physiol.* 169 971–985. 10.1104/pp.15.00636 26297141 PMC4587453

[B41] Luna E. López A. Kooiman J. Ton J. (2014). Role of NPR1 and KYP in long-lasting induced resistance by β-aminobutyric acid. *Front. Plant Sci.* 5:184. 10.3389/fpls.2014.00184 24847342 PMC4021125

[B42] Mackey D. Holt B. F. III Wiig A. Dangl J. L. (2002). RIN4 interacts with *Pseudomonas* syringae type III effector molecules and is required for RPM1-mediated resistance in *Arabidopsis*. *Cell* 108 743–754. 10.1016/S0092-8674(02)00661-X 11955429

[B43] Mari M. Di Francesco A. Bertolini P. (2014). Control of fruit postharvest diseases: old issues and innovative approaches. *Stewart Postharvest Rev.* 10 1–4. 10.2212/spr.2014.1.1 25112557

[B44] Mengiste T. Laluk K. AbuQamar S. (2009). “Mechanisms of induced resistance against *B. cinerea*,” in *Postharvest Pathology*, eds Prusky D. Gullino M. (Dordrecht: Springer), 13–30. 10.1007/978-1-4020-8930-5_2

[B45] Nath J. Watson C. V. (1980). Acid phosphatase changes associated with development of male sterile and fertile maize (*Zea mays* L.). *Biochem. Genet.* 18 377–387. 10.1007/BF00484250 7192554

[B46] Nie P. P. Li X. Wang S. N. Guo J. H. Zhao H. W. Niu D. D. (2017). Induced systemic resistance against *Botrytis cinerea* by *Bacillus cereus* AR156 through a JA/ET- and NPR1-dependent signaling pathway and activates PAMP-triggered immunity in *Arabidopsis*. *Front. Plant Sci.* 8:238. 10.3389/fpls.2017.00238 28293243 PMC5329000

[B47] Nishizawa A. Yabuta Y. Yoshida E. Maruta T. Yoshimura K. Shigeoka S. (2006). *Arabidopsis* heat shock transcription factor A2 as a key regulator in response to several types of environmental stress. *Plant J.* 48 535–547. 10.1111/j.1365-313X.2006.02889.x 17059409

[B48] Noël L. D. Cagna G. Stuttmann J. Wirthmüller L. Betsuyaku S. Witte C. P. (2007). Interaction between SGT1 and cytosolic/nuclear HSC70 chaperones regulates *Arabidopsis* immune responses. *Plant Cell* 19 4061–4076. 10.1105/tpc.107.051896 18065690 PMC2217652

[B49] Nover L. Bharti K. Doring P. Mishra S. K. Ganguli A. Scharf K. D. (2001). *Arabidopsis* and the heat stress transcription factor world: how many heat stress transcription factors do we need? *Cell Stress Chaperon* 6 177–189. 10.1379/1466-1268(2001)006<0177:aathst>2.0.co;2 PMC434399 11599559

[B50] Oh B. J. Ko M. K. Kostenyuk I. Shin B. Kim K. S. (1999). Coexpression of a defensin gene and a thionin-like via different signal transduction pathways in pepper and *Colletotrichum gloeosporioides* interactions. *Plant Mol. Biol.* 41 313–319. 10.1023/a:1006336203621 10598099

[B51] Pajerowska-Mukhtar K. M. Wang W. Tada Y. Oka N. Tucker C. L. Fonseca J. P. (2012). The HSF-like transcription factor TBF1 is a major molecular switch for plant growth-to-defense transition. *Curr. Biol.* 22 103–112. 10.1016/j.cub.2011.12.015 22244999 PMC3298764

[B52] Perazzolli M. Roatti B. Bozza E. Pertot I. (2011). *Trichoderma harzianum* T39 induces resistance against downy mildew by priming for defense without costs for grapevine. *Biol. Control.* 58 74–82. 10.1016/j.biocontrol.2011.04.006

[B53] Pérez-Salamó I. Papdi C. Rigó G. Zsigmond L. Vilela B. Lumbreras V. (2014). The heat shock factor A4A confers salt tolerance and is regulated by oxidative stress and the mitogen-activated protein kinases MPK3 and MPK6. *Plant Physiol.* 165 319–334. 10.1104/pp.114.237891 24676858 PMC4012591

[B54] Pick T. Jaskiewicz M. Peterhänsel C. Conrath U. (2012). Heat shock factor HsfB1 primes gene transcription and systemic acquired resistance in *Arabidopsis*. *Plant Physiol.* 159 52–55. 10.1021/bi981774j 22427343 PMC3375984

[B55] Porat R. Vinokur V. Weiss B. Cohen L. Daus A. Goldschmidt E. E. (2003). Induction of resistance to *Penicillium digitatum* in grapefruit by β-aminobutyric acid. *Eur. J. Plant. Pathol.* 109 901–907. 10.1023/B:EJPP.0000003624.28975.45

[B56] Puttick D. Dauk M. Lozinsky S. Smith M. A. (2009). Overexpression of a FAD3 desaturase increases synthesis of a polymethylene-interrupted dienoic fatty acid in seeds of *Arabidopsis thaliana* L. *Lipids* 44 753–757. 10.1007/s11745-009-3315-5 19548018

[B57] Ramakrishna W. Deng Z. P. Ding C. K. Handa A. K. Ozminkowski R. H. (2003). A novel small heat shock protein gene, visl, contributes to pectin depolymerization and juice viscosity in tomato fruit. *Plant Physiol.* 131 725–735. 10.1104/pp.012401 12586896 PMC166848

[B58] Romanazzi G. Sanzani S. M. Bi Y. Tian S. P. Martínez P. G. Alkan N. (2016). Induced resistance to control postharvest decay of fruit and vegetables. *Postharvest Biol. Technol.* 122 82–94. 10.1016/j.postharvbio.2016.08.003

[B59] Rosales R. Romero I. Fernandez-Caballero C. Escribano M. I. Merodio C. Sanchez-Ballesta M. T. (2016). Low temperature and short-term high-CO2 treatment in postharvest storage of table grapes at two maturity stages: effects on transcriptome profiling. *Front. Plant Sci.* 7:1020. 10.3389/fpls.2016.01020 27468290 PMC4942463

[B60] Samali A. Robertson J. D. Peterson E. Manero F. van Zeijl L. Paul C. (2001). Hsp27 protects mitochondria of thermotolerant cells against apoptotic stimuli. *Cell Stress Chaperones* 6 49–58. 11525243 10.1379/1466-1268(2001)006<0049:hpmotc>2.0.co;2 PMC434383

[B61] Sarig P. Zutkhi Y. Monjauze A. Lisker N. Ben-Arie R. (1997). Phytoalexin elicitation in grape berries and their susceptibility to *Rhizopus stolonifer*. *Physiol. Mol. Plant Pathol.* 50 337–347. 10.1006/pmpp.1997.0089

[B62] Scharf K. D. Berberich T. Ebersberger I. Nover L. (2012). The plant heat stress transcription factor (Hsf) family: structure, function and evolution. *Biochim. Biophys. Acta.* 1819 104–119. 10.1016/j.bbagrm.2011.10.002 22033015

[B63] Sham A. Al-Ashram H. Whitley K. Iratni R. El-Tarabily K. A. AbuQamar S. F. (2019). Metatranscriptomic analysis of multiple environmental stresses identifies RAP2.4 gene associated with *Arabidopsis* immunity to *Botrytis cinerea*. *Sci. Rep.* 9:17010. 10.1038/s41598-019-53694-1 31740741 PMC6861241

[B64] Sham A. Al-Azzawi A. Al-Ameri S. Al-Mahmoud B. Awwad F. Al-Rawashdeh A. (2014). Transcriptome analysis reveals genes commonly induced by *Botrytis cinerea* infection, cold, drought and oxidative stresses in *Arabidopsis*. *PLoS One* 9:e113718. 10.1371/journal.pone.0113718 25422934 PMC4244146

[B65] Sham A. Moustafa K. Al-Ameri S. Al-Azzawi A. Iratni R. AbuQamar S. (2015). Identification of *Arabidopsis* candidate genes in response to biotic and abiotic stresses using comparative microarrays. *PLoS One* 10:e0125666. 10.1371/journal.pone.0125666 25933420 PMC4416716

[B66] Sham A. Moustafa K. Al-Shamisi S. Alyan S. Iratni R. AbuQamar S. (2017). Microarray analysis of *Arabidopsis* WRKY33 mutants in response to the necrotrophic fungus *Botrytis cinerea*. *PLoS One* 12:e0172343. 10.1371/journal.pone.0172343 28207847 PMC5313235

[B67] Shirasu K. (2009). The HSP90-SGT1 chaperone complex for NLR immune sensors. *Annu. Rev. Plant Boil.* 60 139–164. 10.1146/annurev.arplant.59.032607.092906 19014346

[B68] Siegrist J. Orober M. Buchenauer H. (2000). β-Aminobutyric acid-mediated enhancement of resistance in tobacco to tobacco mosaic virus depends on the accumulation of salicylic acid. *Physiol. Mol. Plant Pathol.* 56 95–106. 10.1006/pmpp.1999.0255

[B69] Slaughter A. Daniel X. Flors V. Luna E. Hohn B. Mauch-Mani B. (2012). Descendants of primed *Arabidopsis* plants exhibit resistance to biotic stress. *Plant Physiol.* 158 835–843. 10.1104/pp.111.191593 22209872 PMC3271771

[B70] Sugio A. Dreos R. Aparicio F. Maule A. J. (2009). The cytosolic protein response as a subcomponent of the wider heat shock response in *Arabidopsis*. *Plant Cell* 21 642–654. 10.1105/tpc.108.062596 19244141 PMC2660624

[B71] Swindell W. R. Huebner M. Weber A. P. (2007). Transcriptional profiling of *Arabidopsis* heat shock proteins and transcription factors reveals extensive overlap between heat and non-heat stress response pathways. *BMC Genomics* 8:125. 10.1186/1471-2164-8-125 17519032 PMC1887538

[B72] Tada Y. Spoel S. H. Pajerowska-Mukhtar K. Mou Z. Song J. Wang C. (2008). Plant immunity requires conformational charges of NPR1 via S-nitrosylation and thioredoxins. *Science* 321 952–956. 10.1126/science.1156970 18635760 PMC3833675

[B73] Takahashi H. Kanayama Y. Zheng M. S. Kusano T. Hase S. Ikegami M. (2004). Antagonistic interactions between the SA and JA signaling pathways in *Arabidopsis* modulate expression of defense genes and gene-for-gene resistance to cucumber mosaic virus. *Plant Cell Physiol.* 45 803–809. 10.1093/pcp/pch085 15215516

[B74] Tanabe K. Matsushima-Nishiwaki R. Dohi S. Kozawa O. (2010). Phosphorylation status of heat shock protein 27 regulates the interleukin-1β-induced interleukin-6 synthesis in C6 glioma cells. *Neuroscience* 170 1028–1034. 10.1016/j.neuroscience.2010.08.014 20732391

[B75] Thevenet D. Pastor V. Baccelli I. Balmer A. Vallat A. Neier R. (2017). The priming molecule β-aminobutyric acid is naturally present in plants and is induced by stress. *New Phytol.* 213 552–559. 10.1111/nph.14298 27782340

[B76] Ton J. Jakab G. Toquin V. Flors V. Iavicoli A. Maeder M. N. (2005). Dissecting the beta-aminobutyric acid-induced priming phenomenon in *Arabidopsis*. *Plant Cell* 17 987–999. 10.1105/tpc.104.029728 15722464 PMC1069713

[B77] Ton J. van der Ent S. van Hulten M. Pozo M. van Oosten V. van Loon L. C. (2009). Priming as a mechanism behind induced resistance against pathogens, insects and abiotic stress. *IOBC/WPRS Bull.* 44 3–13. 10.2307/3308631

[B78] Ton J. van Pelt J. A. van Loon L. C. Pieterse C. M. J. (2002). Differential effectiveness of salicylate-dependent and jasmonate/ethylene-dependent induced resistance in *Arabidopsis*. *Mol. Plant Microbe. Interact.* 15 27–34. 10.1094/MPMI.2002.15.1.27 11858171

[B79] Uyttewaal M. Traas J. Hamant O. (2010). Integrating physical stress, growth, and development. *Curr. Opin. Plant Biol.* 13 46–52. 10.1016/j.pbi.2009.10.004 19914123

[B80] van der Does D. Leon-Reyes A. Koornneef A. van Verk M. C. Rodenburg N. Pauwels L. (2013). Salicylic acid suppresses jasmonic acid signaling downstream of SCFCOI1-JAZ by targeting GCC promoter motifs via transcription factor ORA59. *Plant Cell* 25 744–761. 10.1105/tpc.112.108548 23435661 PMC3608790

[B81] van Hulten M. Pelser M. Loon L. C. V. Pieterse C. M. J. Ton J. (2006). Costs and benefits of priming for defense in *Arabidopsis*. *Proc. Natl. Acad. Sci. U.S.A.* 103 5602–5607. 10.1073/pnas.0510213103 16565218 PMC1459400

[B82] Vitrac X. Bornet A. Vanderlinde R. Valls J. Richard T. Delaunay J. C. (2005). Determination of stilbenes (δ-viniferin, trans-astringin, trans-piceid, cis- and trans-resveratrol, ε-viniferin) in Brazilian wines. *J. Agric. Food Chem.* 53 5664–5669. 10.1021/jf050122g 15998130

[B83] von Koskull-Döring P. Scharf K. D. Nover L. (2007). The diversity of plant heat stress transcription factors. *Trends Plant. Sci.* 12 452–457. 10.1016/j.tplants.2007.08.014 17826296

[B84] Vos I. A. Pieterse C. M. J. van Wees S. C. M. (2013). Costs and benefits of hormone-regulated plant defences. *Plant Pathol.* 62 43–55. 10.1111/ppa.12105

[B85] Wang J. Cao S. F. Wang L. Wang X. L. Jin P. Zheng Y. H. (2018). Effect of β-Aminobutyric acid on disease resistance against *Rhizopus* rot in harvested peaches. *Front. Microbiol.* 9:1505. 10.3389/fmicb.2018.01505 30042749 PMC6048224

[B86] Wang K. T. Jin P. Han L. Shang H. T. Tang S. S. Rui H. J. (2014). Methyl jasmonate induces resistance against *Penicillium citrinum* in Chinese bayberry by priming of defense responses. *Postharvest Biol. Technol.* 98 90–97. 10.1016/j.postharvbio.2014.07.009

[B87] Wang K. T. Li C. H. Lei C. Y. Jiang Y. B. Qiu L. L. Zou X. Y. (2020). β-aminobutyric acid induces priming defence against *Botrytis cinerea* in grapefruit by reducing intercellular redox status that modifies posttranslation of VvNPR1 and its interaction with VvTGA1. *Plant Physiol. Biochem.* 156 552–565. 10.1016/j.plaphy.2020.09.026 33059266

[B88] Wang K. T. Li C. H. Lei C. Y. Zou Y. Y. Li Y. J. Zheng Y. H. (2021). Dual function of VvWRKY18 transcription factor in the β-aminobutyric acid-activated priming defense in grapes. *Physiol. Plantarum* 10.1111/ppl.13341 [Epub ahead of print]. 33483982

[B89] Wang K. T. Liao Y. X. Xiong Q. Kan J. Q. Cao S. F. Zheng Y. H. (2016). Induction of direct or priming resistance against *Botrytis cinerea* in strawberries by β-Aminobutyric acid and their effects on sucrose metabolism. *J. Agr. Food Chem.* 64 5855–5865. 10.1021/acs.jafc.6b00947 27368357

[B90] Wang K. T. Wu D. Z. Bo Z. Y. Chen S. Wang Z. R. Zheng Y. H. (2019). Regulation of redox status contributes to priming defense against *Botrytis cinerea* in grape berries treated with β-aminobutyric acid. *Sci. Hortic.* 244 352–364. 10.1016/j.scienta.2018.09.074

[B91] Wang W. X. Vinocur B. Shoseyov O. Altman A. (2004). Role of plant heat-shock proteins and molecular chaperones in the abiotic stress response. *Trends Plant Sci.* 9 244–252. 10.1016/j.tplants.2004.03.006 15130550

[B92] Windram O. Madhou P. McHattie S. Hill C. Hickman R. Cooke E. (2012). *Arabidopsis* defense against *Botrytis cinerea*: chronology and regulation deciphered by high-resolution temporal transcriptomic analysis. *Plant Cell* 24 3530–3557. 10.1105/tpc.112.102046 23023172 PMC3480286

[B93] Xiao S. Chye M. L. (2011). Overexpression of *Arabidopsis* ACBP3 enhances NPR1-dependent plant resistance to *Pseudomonas syringe* pv tomato DC3000. *Plant Physiol.* 156 2069–2081. 10.1104/pp.111.176933 21670223 PMC3149925

[B94] Xin H. B. Zhang H. Chen L. Li X. X. Lian Q. L. Yuan X. (2010). Cloning and characterization of HsfA2 from Lily (*Lilium longiflorum*). *Plant Cell Rep.* 29 875–885. 10.1007/s00299-010-0873-1 20499070

[B95] Yu D. Q. Chen C. H. Chen Z. X. (2001). Evidence for an important role of WRKY DNA binding proteins in the regulation of NPR1 gene expression. *Plant Cell* 13 1527–1540. 10.1105/TPC.010115 11449049 PMC139550

[B96] Zarate S. I. Kempema L. A. Walling L. L. (2007). Silver leaf white fly induces salicylic acid defenses and suppresses effectual jasmonic acid defenses. *Plant Physiol.* 143 866–875. 10.1104/pp.106.090035 17189328 PMC1803729

[B97] Zheng X. Krakowiak J. Patel N. Beyzavi A. Ezike J. Khalil A. S. (2016). Dynamic control of Hsf1 during heat shock by a chaperone switch and phosphorylation. *Elife* 5:e18638. 10.7554/eLife.18638 27831465 PMC5127643

[B98] Zimmerli L. Métraux J. P. Mauch-Mani B. (2001). β-aminobutyric acid-induced protection of *Arabidopsis* against the necrotrophic fungus *Botrytis cinerea*. *Plant Physiol.* 126 517–523. 10.1104/pp.126.2.517 11402183 PMC111145

