# Corrigendum: Heat Shock Protein HSP24 Is Involved in the BABA-Induced Resistance to Fungal Pathogen in Postharvest Grapes Underlying an NPR1-Dependent Manner

**DOI:** 10.3389/fpls.2021.812672

**Published:** 2021-12-08

**Authors:** Chunhong Li, Shifeng Cao, Kaituo Wang, Changyi Lei, Nana Ji, Feng Xu, Yongbo Jiang, Linglan Qiu, Yonghua Zheng

**Affiliations:** ^1^College of Life and Food Engineering, Chongqing Three Gorges University, Chongqing, China; ^2^College of Food Science and Technology, Nanjing Agricultural University, Nanjing, China; ^3^College of Biological and Environmental Sciences, Zhejiang Wanli University, Ningbo, China; ^4^College of Food and Pharmaceutical Sciences, Ningbo University, Ningbo, China

**Keywords:** β-aminobutyric acid, heat shock protein, priming resistance, NPR1, *Botrytis cinerea*, grape berries

In the original article, there was an indeterminacy in Figure 5A as published. The figure depicted a small white dot of a single colony or a little colony cluster presented on the SD-T-L-H plate. We carelessly thought the white dot to be a bubble from the process of pouring the synthetic dropout medium into Petri dishes at first, because the single colony was extremely similar to a bubble. However, the presence of an actual single colony or colony cluster in SD-T-L-H plate might be caused by the self-activation of the “bait” pGBKT7 plasmid, thus leading to the “false positive” image in Y2H experiments with a very low probability (<5%). Of course, the result of His pull-down exhibited an obvious interaction between VvHSP24 and VvNPR1 *in vitro*.

**Figure 5:**
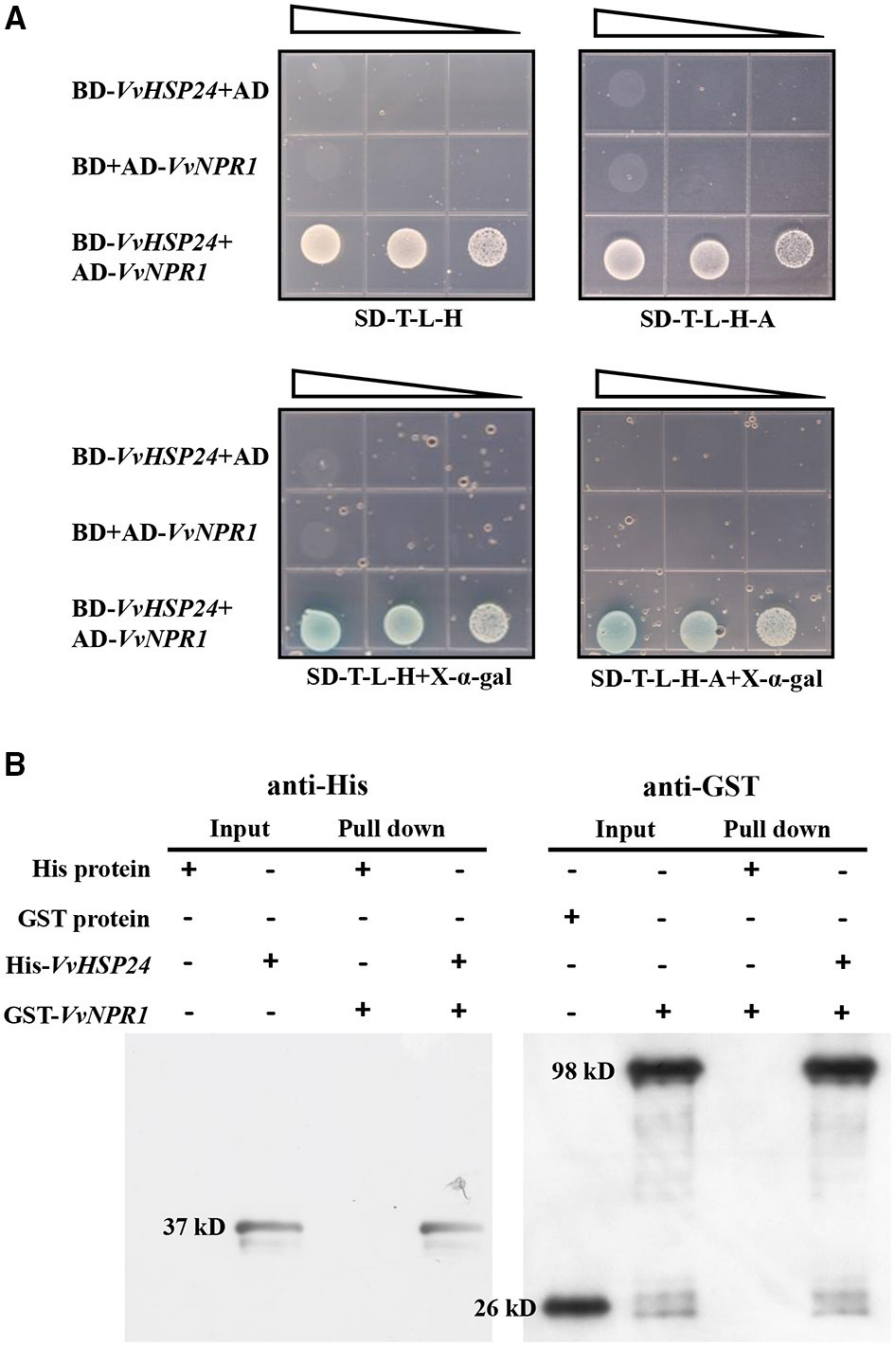
*VvHSP24* interacts with *VvNPR1 in vivo* and *in vitro*. **(A)** Yeast two-hybrid analysis of the physical interaction between *VvHSP24* and *VvNPR1*. SD-T-L-H, SD/-Trp-Leu-His agar medium; SD-T-L-H-A, SD/-Trp-Leu-His-Ade agar medium. The right-angled triangles on the top of the gridded Petri dishes represent the absorbance of yeasts at 600 nm in a 10-fold dilution series, from 1 to 10^−2^ abs. **(B)** The GST-fused *VvNPR1* protein (1 mL) was incubated with 1 mL of preimmobilized His-*VvHSP24* protein in a total volume of 25 mL at 4°C for more than 8 h. The pulled down proteins (6 μL) were analyzed by western blotting with anti-His or anti-GST antibodies.

In past month, we re-conducted the Y2H with three replications following the method as described in the published M&M to completely confirm the interaction. Meanwhile, pull-down and co-IP were both done in this period. The obtained Y2H results showed that the colony clusters on SD-T-L-H and SD-T-L-H-A plates with or without X-α-gal were absolutely caused by the physical interaction between VvHSP24 and VvNPR1, but not the self-activation of the pGBKT7 vector, because no colony appeared among the negative controls (BD-VvHSP24 + AD and BD + AD-VvNPR1) in dropout plates. The pull-down and co-IP assays confirmed the interaction between VvHSP24 and VvNPR1 *in vitro* and in plant cells, which were consistent with the representative result of Y2H.

The correct Figure 5 appears below.

The authors apologize for this error and state that this does not change the scientific conclusions of the article in any way. The original article has been updated.

## Publisher's Note

All claims expressed in this article are solely those of the authors and do not necessarily represent those of their affiliated organizations, or those of the publisher, the editors and the reviewers. Any product that may be evaluated in this article, or claim that may be made by its manufacturer, is not guaranteed or endorsed by the publisher.

