# Supplementary material for: Heat Shock Protein HSP24 Is Involved in the BABA-Induced Resistance to Fungal Pathogen in Postharvest Grapes Underlying an NPR1-Dependent Manner
Source: Front Plant Sci. 2021 Mar 8;12:646147. doi: 10.3389/fpls.2021.646147 (PMC7984168; doi:10.3389/fpls.2021.646147)
Supplement: Supplementary Figure 1 — Tree analysis of the isolated Botrytis cinerea strain with the Botrytis cinerea family. Accession numbers of sequences: Botrytis cinerea B05.10, MH427722.1; Botryotinia fuckeliana T4, AJ422103.1; Botrytis cinerea Bo.CC166, MH427722.1; Botrytis cinerea strain B23, AJ422103.1; Botrytis cinerea BOT 40, KU145363.1; Botrytis cinerea BOT 5, KX772771.1; Botrytis cinerea BOT 16, KX781162.1; Botrytis cinerea BOT 61, KX781165.1; Botryotinia fuckeliana strain Minhang, AY694146.1; Botrytis cinerea BOT 74, KX781167.1; Botrytis cinerea strain SCB7-5, KR080285.1; Botrytis cinerea Bot 68, KU145391.1; Botrytis cinerea strain SCB2-2, KR080283.1; Botrytis cinerea strain SCB7-4, KP165498.1; Botrytis cinerea strain SCB5-2, KP165495.1; Botrytis cinerea strain BC-8, KP141792.1; Botryotinia fuckeliana species; AY674786.1; Botrytis cinerea strain KBC-13, KP141795.1; Botrytis cinerea strain SL, KU936083.1; Botrytis cinerea strain BC-10, KP141793.1; Botrytis cinerea strain KBC-14, KP141794.1; Botrytis cinerea strain BC-1, KP141790.1; Botrytis cinerea strain AC1, KU936079.1; Botrytis cinerea strain AR, KU936082.1; Botrytis cinerea strain SV, KU936085.1. [file Data_Sheet_1.doc]

**SUPPLEMENTARY MATERIAL**

**FIGURE S1**

**FIGURE S2**

**FIGURE S3**

**FIGURE S4**

**FIGURE S5**

**
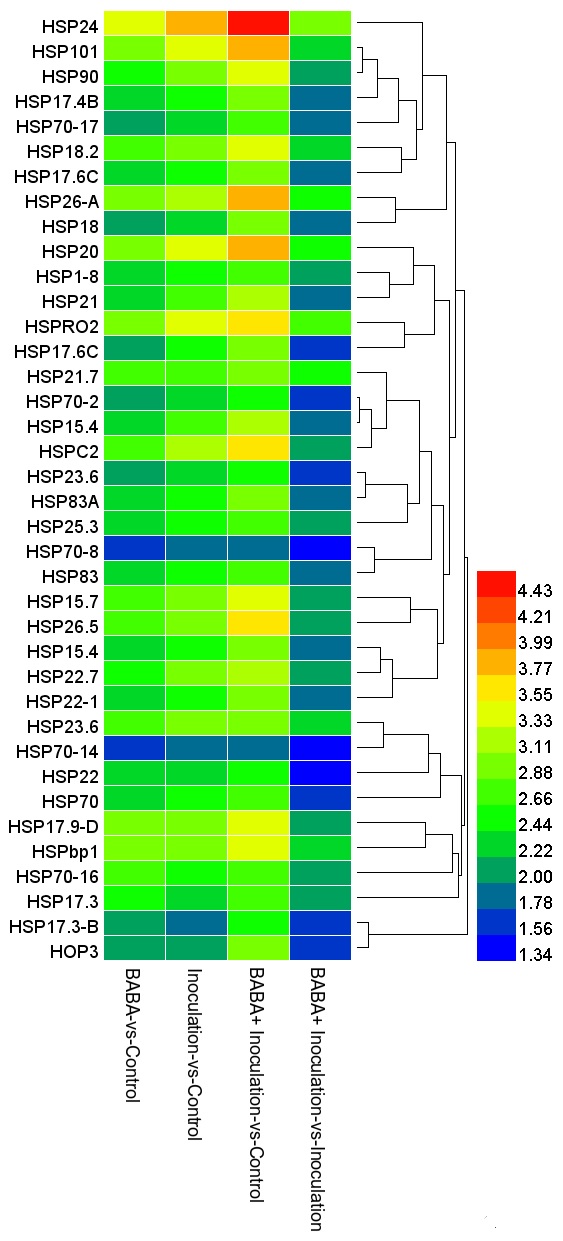
**

**FIGURE S6**

**
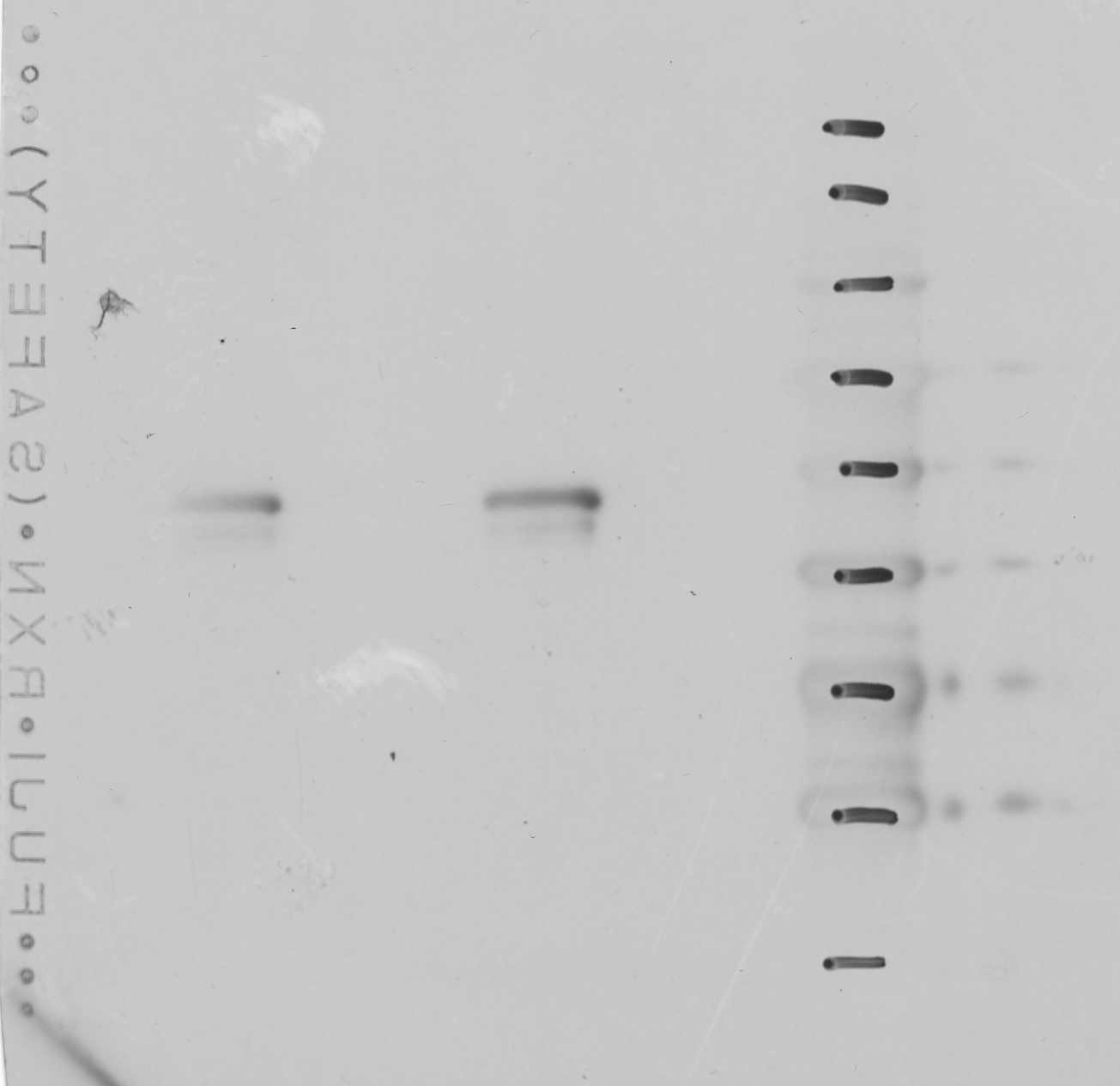
**

**FIGURE S7**

**
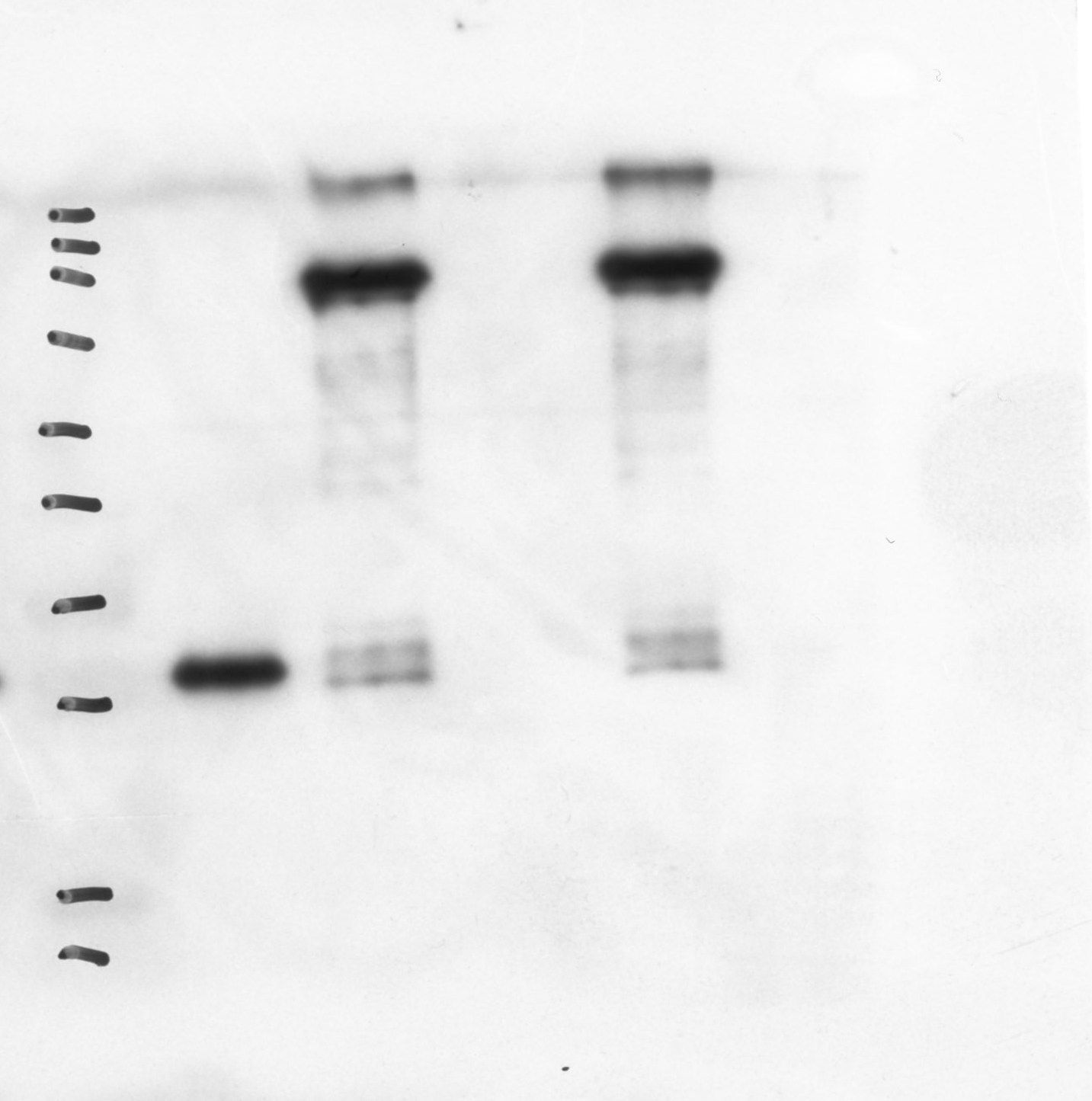
**

**FIGURE S8**

**
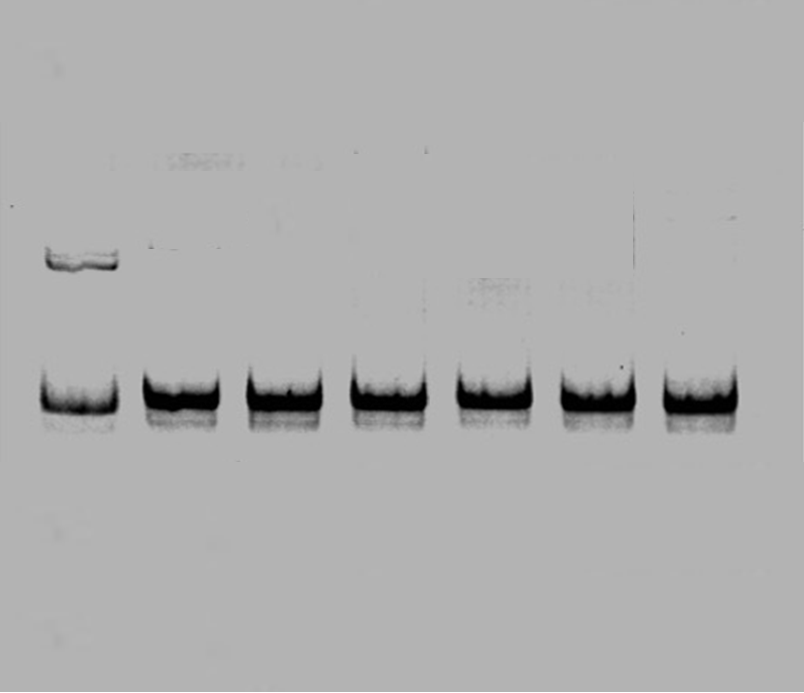
**

TABLE S1

| **Gene ID** | **Primer name** | | | **Oligonucleotide primers** | **Purpose or vector** | |
| --- | --- | --- | --- | --- | --- | --- |
| LOC100232866 | *VvActin7*-qRT-PCR-F  *VvActin7*-qRT-PCR-R | | ACAATGAGCTTCGGGTTGCC  GCAACATACATGGCAGGCACA | | | qRT-PCR |
| AT3G18780 | *AtActin2*-qRT-PCR-F  *AtActin2*-qRT-PCR-R | | TTGTTCCAGCCCTCGTTTGT  TTTGCTCATACGGTCAGCGA | | | qRT-PCR |
| LOC100247320 | *VvHSP24*-qRT-PCR-F  *VvHSP24*-qRT-PCR-R | | TTTGGGGTTTGGCTGAAGGG  GACCTTGCTGGTCATCCACG | | | qRT-PCR |
| NM_119862.3 | *AtHSFB1*-qRT-PCR-F  *AtHSFB1*-qRT-PCR-R | | GGCGGGGAAATGTGTTGTTG  GCAACCATGTTCTCCACCGA | | | qRT-PCR |
| LOC100250996 | *VvNPR1*-qRT-PCR-F  *VvNPR1*-qRT-PCR-R | | GCTGATGCCCAGAGGACAAC  AAGCGTTTCCCAAGGTCCAC | | | qRT-PCR |
| LOC100258414 | *VvPR1*-qRT-PCR-F  *VvPR1*-qRT-PCR-R | | GCACTCCTTTGTTTCATGGGGT  CAGCCACAGTGTTGTCCCAT | | | qRT-PCR |
| LOC100232892 | *VvPR2*-qRT-PCR-F  *VvPR2*-qRT-PCR-R | | TCAGCATGTCAAGGGTGGGA  AGAGCCCCCAGTGTTTCTCAT | | | qRT-PCR |
| LOC100254811 | *VvPR5*-qRT-PCR-F  *VvPR5*-qRT-PCR-R | | AATCACAGATGCCCCAAGGAC  TGGCTCGCAGGTTTCAAGAG | | | qRT-PCR |
| LOC100257377 | *VvPDF1.2*-qRT-PCR-F  *VvPDF1.2*-qRT-PCR-R | | TTGGAAGCAAAGGTCTGCCA  CACCCTCCCAGTTCTTGCAC | | | qRT-PCR |
| LOC100244898 | *VvVSP1*-qRT-PCR-F  *VvVSP1*-qRT-PCR-R | | GCCATGTGCAGATCGTGTTG  GGCAGGGCTTTATGGCGTT | | | qRT-PCR |
| LOC100260476 | *VvHEL*-qRT-PCR-F  *VvHEL*-qRT-PCR-R | | CTACTGCGCCACTTGGGATG  TGTGTCCCCGTGCCAGTATT | | | qRT-PCR |
| LOC100257229 | *VvFAD3*-qRT-PCR-F  *VvFAD3*-qRT-PCR-R | | TGGCTATGTGCTTGCAGACC  ATGATCGTCCAGGGCCACTT | | | qRT-PCR |
| LOC100256715 | *VvTHI2*-qRT-PCR-F  *VvTHI2*-qRT-PCR-R | | TGCAAGCTCGGATGTGCTTC  ATGGGTGGTACTCGGTGCAT | | | qRT-PCR |
| LOC100241730 | *VvERS1*-qRT-PCR-F  *VvERS1*-qRT-PCR-R | | ATAGCGCGGAAGCAATACGG  ACCCGGACAGCAACAACATC | | | qRT-PCR |
| LOC104882330 | *VvERF1*-qRT-PCR-F  *VvERF1*-qRT-PCR-R | | ATGAGCTGGGGTGTTATGGC  TCCTTGGCAGAGAGAACCACT | | | qRT-PCR |
| AT4G26120 | *AtNPR1*-qRT-PCR-F  *AtNPR1*-qRT-PCR-R | | GTCGGCTTTGACTCGGTTGT  TCGTCTACGCAAGCAGAAGC | | | qRT-PCR |
| AT2G14610 | *AtPR1*-qRT-PCR-F  *AtPR1*-qRT-PCR-R | | GTTCACAACCAGGCACGAGG  AGTTTTCCCCGTAAGGCCCA | | | qRT-PCR |
| AT3G57260 | *AtPR2*-qRT-PCR-F  *AtPR2*-qRT-PCR-R | | ACGTCTCCTCCGTCTCAAGG  AGGGAGATTGCTTGCTTGCC | | | qRT-PCR |
| AT1G75040 | *AtPR5*-qRT-PCR-F  *AtPR5*-qRT-PCR-R | | ATTGCTGCCGTGGAGCTAAC  GGTGCTCGTTTCGTCGTCAT | | | qRT-PCR |
| XM_002277729.4 | *VvHSP24*-SpeI-F  *VvHSP24*-BamHI-R | | GGACAGCCCAGATCAACTAGTATGGCACAAAGATCGGTTCCG  GCCCTTGCTCACCATGGATCCGTTGCAGACCTTGCTGGTCATC | | | Subcellular localization |
| XM_002277729.4 | *VvHSP24*-SmaI-F  *VvHSP24*-BamHI-R | | CATGGAGGCCGAATTCCCGGGATGGCACAAAGATCGGTTCCG  CCGCTGCAGGTCGACGGATCCGTTGCAGACCTTGCTGGTCATC | | | pGBKT7/Y2H |
| XM_002281439.4 | *VvNPR1*-SmaI-F  *VvNPR1*-BamHI-R | | GGCCAGTGAATTCCACCCGGGATGGACTACAGAGCTGCTCTCTCC  CAGCTCGAGCTCGATGGATCCCTTCTTGCAAGAGAGTCTACTGTTATTCC | | | pGADT7/Y2H |
| XM_002281439.4 | *VvNPR1*-NotI-F  *VvNPR1*-BamHI-R | | TCAGTCAGTCACGATGCGGCCGCATGGACTACAGAGCTGCTCTCTCC  GATCTGGTTCCGCGTGGATCCCTTCTTGCAAGAGAGTCTACTGTTATTCC | | | Fusing GST/ pGEX-4T-1 |
| XM_002277729.4 | *VvHSP24*-NotI-F  *VvHSP24*-BamHI-R | | TGGTGGTGCTCGAGTGCGGCCGCATGGCACAAAGATCGGTTCCG  CAGCAAATGGGTCGCGGATCCGTTGCAGACCTTGCTGGTCATC | | | Fusing His/ pET-28a |
| XM_002277729.4 | *VvHSP24*-SpeI-F  *VvHSP24*-BamHI-R | | GGACAGCCCAGATCAACTAGTATGGCACAAAGATCGGTTCCG  GCCCTTGCTCACCATGGATCCTCAGTTGCAGACCTTGCTGGT | | | pCambia 1305.1/heterologous overexpression |
| XM_002277729.4 | *VvHSP24*-XbaI-F  *VvHSP24*-SacI-R | | CTCTAGAATGGCACAAAGATCGGTTCCG  GCGAGCTCTCAGTTGCAGACCTTGCTGGT | | | Fusing His/ pCzn1 |
|  | M2CRISPR-T1-F  M2CRISPR-T2-R | | ATATATGGTCTCGATTGCACAGACGACGTCGTTTCAGTTTTAGAGCTAGAA  ATTATTGGTCTCGAAACTCATACGGGACCCACTCACCCAATCTCTTAGTCGA | | | M2CRISPR |
|  | HygF  HygR | | GCTTCTGCGGGCGATTTGTGT  GGTCGCGGAGGCTATGGATGC | | | Identification of hygromycin marker gene |
|  | *AtHSFB1*-CRP-F  *AtHSFB1*-CRP-R | TGTTGGTTCGCCTTCTGAGT  TCTCCTTTCAACCACACCCC | | | | Sequencing to identify mutations |

**DATA S1**

*VvPR1* (LOC100258414) promoter:

CTTTAAAATTGTTTGTTTAAAAGAAAATTCTCAAAATTTAGCTCTTAAACTAAAGTTTTCTTTGAAAAATTAATTTTCTATTTTACTCATTTCCACCCTAATAGATTTGAAAATTTATATTATATTGAAGTTGCATTTTCACCGCAAAATTATTATTTTTTTATTTCTATAAATAAGCAAAAATTTCATTGTTAAAATTTTCAAAAGTAATATTTTATTTGATACTTTCCTATAGAAATAAATTTTAGAATTTTTTTTTGAATTATGACATTTTAAAAAGAAATTTTGAAAAATATTGTTTCTTTTCTCTTTAAATCCCATCAAAGTTGATAGTCTAGTAGCTGTGAATTTATCCTTGATCAGAGTTCTGATTTTGGAGCTTCATGCCCCAATGAAATAAATAAGAAATATAATTTTGATACTAAGAGCAACAATTGGCAAGCTATATATATTTTGAAATTATTGAGAAGAATCCGATATTATCAACCCATCCTTGACATAAGATTTGTCTCAACTTTCTTGCTTAAATGAAACAAACAAGAAAAGTAAATTAAGAATATGTTCTTGTTTTTAGTAAAAATATTTTTAATAAAAATGTTTTTAAGAAAATCATCGATCAAGTGTTTATCCGAAACACATCATAAGAGATTTTTTAACATTATAAAGTAACTTTTTAGATTTTAACAAGTGTTTTTTAAAATTTTATTAAACACCCAACTTGTTTTTCTCCTTAAAAATACCTTCTGGAATCACTGAGATTCAATCCTAAGTCACTCCAAGTGTTTTTTAAAAGCACTATCAAACGAGTTTTAAATCAAAGATAATACTGGAACATCAATTATTATTATTATATTGATAAATTATCATATTAATTAAGGTCATTTGAAGATATTTTTAGATGATTAATGCGTTTGACAATGATTTTAATAAATATTTTTAACATTTTTAATACTTTAAAAAATTTTATCTTTCAAATATTAAAAATATTTAAAACGCTTCATAAAATCATTATCAAATATACGGGACAAATTAAGTTTTTAAGAATGAGTGAAATAATCATTTATTGAAAATTTTAACTCCAGGCTTTCTCACCAACAAATCAAATATGAAAAGATATAAAATTTTTATTATTACACATGTCTTAATCATACTGTTTTGGCGGCTTTGGATTTACAACTGTCTTTTACGAGAGGGAATAAGGGATATTTTATTATTTTTTCCATTCTTGTATGATAAAAAATAATTTAAATGACAATAAAAGTGGAATAAAAAATAATTTCTATAATAATAAAATTCCACTTATAAGTTAAATTTGAATTAATCTTGTAAAGGAGGCTATCGAAACGATATATAATATCTTAAAACTGCTTATTTATCCCTATATTTTCATTAATTTTTTATTATTTTTATGATTAATTTTTATTTATTAAGTAAACATTAATTTTTTAATCTTATTATTTAGTAAAAAAAATGTTTAAATGATGTTCTTTTAAAATAAAAATAAAAATTTAAATTATATATAAGAATATTATTATCAATTTATTTATTTATTATTTTCATTCATTTTTCAATTATTACAAAATATTCAAGTATAAACAAAGAATTATTTTAATCTTTCATTCTTAGTGCATTTGAAGATAAGAATCGAAATCATCAAATTTATTTTTACTTTCTTTAAGTATGAGAATAGAAACAAAATATTAATTTTCATTATTTATTCTTATCCATTATGATATAGGTGTAAAAGATATCATACATAGTCTAAAAGGAATCTGACACAAGAATGAAATTCAAATAATGTTGATTCATTTATCCTTTTAGAAGAAAAGTAGTCTTGATAAAACTGGTATGGCCCCATGAGTTACAAAAAGAAGAAATTTCAAATAATGTTGATTCATTTATCCTTTTAGAAGATAAGTTGTCTTGATAAACTGGTATGGCCCGCATGAGTTACAACAAGAAGAAATTTCAAATATTGCTCTCCGTGGAAGCCATTTCATCAGTAGTGTCCTTTTACGTGTGAAAACTGGTCCAAGGGGGCGTTTGGTGGAAATG

*VvPR2* (LOC100232892) promoter:

CGAAAAGAACCAATTTTATTATTACTCATCTCAATATCAATATTTAAGAACTTTCTAATAAAATCATCCTTATCCGGAAATCAAATTGGATTTTGGAGTTTTATTTGTCAGTTGTCACACCATGATTTGCGCTCGTCACCAAGTGCAGCCAGTTGGGTCAAGTCCCACTCCAAGCCTATCAAGATAGACTAAGTCATTGAACCAATTGTAATTTGATAAATTTATGATACCCACTTCTTAAAGCTTCAAATTTCCACATACCATCACTCAAATCCAACGGCCTGGAAAATGTTTTCCATCAACGCACATTGTTTGCTAAGAGTGGACAAGGGGAATTTCCACATGGATAGTGACTTCTTCTGCTGCCCATGTGCAAATTATGTGGCTAATCTTGTTTGAAATAGGGGCAATGATATGGTTTGGCAATTGGCATTTTATGTGGCTAATGTTTTGGTCTTGGTCATCACCCTCTCCACACTTGCATTTAGGAACCTCGTTGCTCAAGTCTTTGCTGCCTTACTTTTCTTGATTAATAATATTGAAGATTTACTAAATAAGTATTATTTTTATATTACTCCTTTTTTTTTTCTTTTCTTTTTGAAAAGGGATAAGAAAACGGAAGGGAAGTGGGACTCATTATTTATATATATAATTAGACAAAAATAAAAATGAAAAAAAAATCCCTTCGCTACCCTAATTCATACCATGATTATATATTAGGATTATTTAATTAAAATGATTATTAAAAACTCAAATTTTAAAATTTAATCCTTTATTTTTTTTGGTTTCATATTGATAAAAATCTTCTTATATTTTCTTCTAAAAATATTTTTTTTAAATATAATATAAATACTTGAAATAGTCAATGATATTTATATTAAAAATGAGTTAATCTTCAAATAAAAATATGAAAAAAAAAATAAATTTATAAACATGAGAAGTATTTCATCATTTTAATAAATTATTTCTATGTATTATTATTATTAGGTAATTTTATATTTATAATGCTTTAAAAAATAATATATTATATATTATTTTATATTCAAAATTTAAAAATTGAATATACAATTGTGACATTAAAAATATTAATAGGAGTAAATTTTTTAATTCCTTTATTTTTCATGCCGTGCGTGCCGTTTAGTCTAAGTTGGAGTAGACGGAGAGTGGGACTCGGATGGGGAAAAATCATCAAAAGGGAGGTGTTTACATTAGAACCAGAAAGATCTCGGATTAAGTTATTTGACTATTCATCCCATTACCTCACATCCGCGTACGAAACAAGATGTAAGGGTCTGATATTTTCGTGCGGCCCAAAAGGAGCCTAGTGTTTTATATTTACCTTTATTTGAAGTGGGTCAGAAACTCAGATTAATCCCAATTTAATGTGACCTGGAAGTTGCATTTAAGTATCTTAAGTTGCTGTACATTTGAGCACAGTGCATTTGATGGTGAATTTAATGTGACCTGAAAGTTGCAATCTTTTAAGTTATCGTGATTTTAAAAAATGTTTTTAATATTTTTAATAATTTTTAAAAAAAAATTAAAATTATTTTTTAAAATTATTGTGAAACATATTTTAAGAGTGTATTTAATAGTAATTTTAAAAAGTATTTTTTATTATTTTTAACACTTAAAAAATAAAAAATTTTAAATATTAAAAAAATTAAAAACACTTTTTAAAATCATTATATAAAACATATTCTTAAAGTTCATAGTGGAAGCTCTAAAGTAATTGGGATTCAATGAACCTAGCTAAAAATTGCATTTCCCCTTCCTAAAAGAAGTGAAATCCAGAGGATAGAGAATTATTAACTTCAATATGAGGATGTAATGTAGGACTTTTCAAAGGGGAGAAAAGTCTCAGAAAACAGACAGAATGGCACTTAGTAGTGCACGTACAAGCTGGTTGGGGAGGTGGATTTATTTATTTATGTAATCTTCTCTCAAATTTCCATGGAGTGCTTGACTTGCGAGGTGGAGACTAGCATTATCATCACTTAAAATTCTGGGTGCATGCGTCCATCCACTGTCTATAAAAGGGAGCTAAATCTCGGTGTTAATGCATACAACCTAGCTTCTTATTTTTCTCTTTCTTCTCCATCATG

*VvPR5* (LOC100254811) promoter:

GTCTCTACAAAGATATGTGGTAGATGGAAAATAGGAACTGATTCCCTAAAACAGAAAAGAATGTATGAACCTTCCTAAAATACATCAATTACAATAATAAAATTGATTCGACTTCCTAAACTTGCCACCAGAACCAGATGTCTCAAACCTCCCCTACTTGCAAGCAGTTGTGAAAGAAACACTTAAGACTACCTTCCATGACCTTTGACAAGGAGAAACACTGCAAAATTTTGTATATTGATTTGAATGATGAGTCTCTACAAAGATATGTACAGTTAAAGTAGATGGAAAATAGGAACTGATTCCCTAAAATCTGTCATGAAGATCAGGAAATAATATCTCAAAAAAATAAAAACAAAAAAGAAGATATGAGCCTTCCTAAAATATATCAATTGCAGTAATAAAATTGATTTGACTTCCTAAACTTGCTGTCAGAACCAAATATCTCAAACCTCCCCTACTTGCAAGCAGTTGTGAAAGAAACACTTAGACTGTACCTTCCATGACCTGTGACAAGGAGAAACACAACAAAATTATGTAATTAGTTGATATATACCAGAAAAAACTGCAGTAGCAATCAATCTCTATGCTAGTATGAAAGACCGTGAGAAATGGGATGATCCTAATGAGTTCCGCTAGAGGTGTTCTTGGTTTCCTCAGAAGAACAAGATGATTTTGAACAAAAACTAAAGAAAAAACATCAATTTTGTCCCATTTGGCAACGTGAGGAGAGGCTGCCCAGTGACATTGTTGGTATCCAGTTTGATGCATACTATGGTTGCTACTGTTCAATGCTTTGATTAGAAGGCTGGTGGAGTTGGACATGGGGCTTAGTTGATATGCACTCTAGCCAGGCATGTCCTTGAGCATGGTTCACTCATTCATGTGTCATCCCATAGTTAACTGTAACCCTTTGGAGGGTCATATCTGGGTATTTGATTCTACAACAAATGCTATTATCATCTATGTCCCAAGTGTTTCAATGCTCTCTAGCTATATCTTAACAGTTGTTCCATGACTAAGAATGAATTGTATATAACAAGACATAGTTTCTCATTGTATGTTATTGGATGATCAGCCAAGGCTGACTATAGGACTCCAGAGCCTCTGCCACGAATTGTATCACCTAGTTGGAAACCAATAGTCATTCCATAAGTCTATTGATTCGCCATTTCCCACAAGGTTCCAGGGAAAAGACAATAATTTAAACCAACTTTGGAAACCCTTAATGTTCATTGTCTAGAGTTAGAAACAACCTTTTGTTTTAAAAAAGGTCGTTATCAGGATTTATATGATACAATAAGTAAAGCTCTTTAACCTGAGGCCAATATTACAGTTAAATAGAACAAGCAGTGCTTACGAAAGGAGTAGAACCAAAACAAATTTGAAAAGCTCTTCCAATCAAATTGATGACTCCATTACACCTAAATTGTTTGCAAACATTTATTTCCCTATTGGTCTAAAGACAAAAGCATGCCACATGGGGTTTTGTCCTCAATAGAGATGGGGATTCAATCTTTTGTACAAAACAGAAAGACTTCAGCAAGATGGGCTAATCATTAAAGCCTAAACATTCTAAAAACATTATATCCCAGATACCTTGACTTAAAACTGCACATTCTTTGAAACAACAGGCATTAGAAGGTTACAATTCTAAATTTAAAAAGGACATCTGATTTTCTGCCAAGTGCATGGTTAAAAATATTGAATTCAAGCCAAGACAACCCTAAAAAATTCAACAACCAAAGGCAGGCCAGATGGCAACAGATCACCTCTCTAAACATCCATTTCTAAAGGGAAAATCCTAGGCTTCTTTTGCTGTTCTAAATAACAAGATCTGGCTAGCCACTTTGATTAGATCAAGTACACTAAATTCAGGGCAGTTTTGACCCTCAAAAGAGAATCTAGGTAATCCGTTATTTTATATCACTGTGAAAAACAGAACACATATCATAACAGAGATTCACCCCCACACCTTAATTTAGAAAACTAGCTCACCCCCCTTTTAAGGATGGTGTGTTGCTGATCCTCCCCCTATATAAATCTCGAGTCCTTTTTTTTAATTGAGACGCCAAATTCCACAACCTAAATTCAGTACTCTCCAATGGCATTGTCATATATTCACCTTGTCTTGGTTTTCTGTGTCTTTGGAACTAGTAGATACACTAATTAACTTTCCATTTCCTGTGACACTTGGTTGATCTACACATCTAAATACAAGTAGTAATGTTGTATTATGTTCTTTATTCTTCTCAGGTGTTGATCCTGCGATTTTCACATTACCAAATAGATGAAGGAAAACTATATGGTCGGGGATCACACCTGGATCTGGAAAACCTCAACTAATG
